# Enteropathogenic *E. coli* infection co-elicits lysosomal exocytosis and lytic host cell death

**DOI:** 10.1128/mbio.01979-23

**Published:** 2023-12-01

**Authors:** Raisa Shtuhin-Rahav, Aaron Olender, Efrat Zlotkin-Rivkin, Etan Amse Bouman, Tsafi Danieli, Yael Nir-Keren, Aryeh M. Weiss, Ipsita Nandi, Benjamin Aroeti

**Affiliations:** 1 Department of Biological Chemistry, The Alexander Silberman Institute of Life Sciences, The Hebrew University of Jerusalem, The Edmond J. Safra Campus–Givat Ram, Jerusalem, Israel; 2 Department of Cell and Developmental Biology, The Alexander Silberman Institute of Life Sciences, The Hebrew University of Jerusalem, The Edmond J. Safra Campus–Givat Ram, Jerusalem, Israel; 3 The Alexander Grass Center for Bioengineering, The Hebrew University of Jerusalem, The Edmond J. Safra Campus–Givat Ram, Jerusalem, Israel; 4 The Protein Production Facility, Wolfson Centre for Applied Structural Biology, The Hebrew University of Jerusalem, The Edmond J. Safra Campus–Givat Ram, Jerusalem, Israel; 5 Faculty of Engineering, Bar Ilan University, Ramat Gan, Israel; UCLA School of Medicine, Los Angeles, California, USA

**Keywords:** enteropathogenic* E. coli*, type III secreted effectors, EspZ, Map, EspF, cell death, lysosomal exocytosis, membrane repair, host-pathogen interactions

## Abstract

Enteropathogenic *Escherichia coli* (EPEC) is a primary human enteric bacterial pathogen causing acute diarrhea in children. EPEC colonizes the small intestine, and the disease is induced, in part, by the ability of the pathogen to utilize a type III secretion machinery to inject a battery of proteins, termed “effectors,” from the bacterial cytoplasm into the intestinal enterocytes. Host cell responses to the infecting pathogen are also essential for disease development. Despite intensive research, the mechanisms of EPEC infection and host cell responses need to be better understood. Here, we show that specific EPEC type III secreted effectors, EspF and Map, induce lytic host cell death and also lysosomal exocytosis (LE), resulting in the secretion of lysosomal enzymes into the extracellular environment and the appearance of the lysosomal membrane proteins, Lamp-1, on the infected cell surface. The mitochondrial cytotoxicity and the guanine nucleotide exchange factor domains of Map have been identified to be involved in these processes. In contrast, EspZ, an EPEC effector that protects against lytic cell death, also inhibits LE. Our results combined suggest that LE and host cell death are tightly interconnected processes. The mechanisms and functional significance of these processes on EPEC infection are discussed.

**IMPORTANCE:**

Enteropathogenic *Escherichia coli* (EPEC) infection is a significant cause of gastroenteritis, mainly in children. Therefore, studying the mechanisms of EPEC infection is an important research theme. EPEC modulates its host cell life by injecting via a type III secretion machinery cell death modulating effector proteins. For instance, while EspF and Map promote mitochondrial cell death, EspZ antagonizes cell death. We show that these effectors also control lysosomal exocytosis, i.e., the trafficking of lysosomes to the host cell plasma membrane. Interestingly, the capacity of these effectors to induce or protect against cell death correlates completely with their ability to induce LE, suggesting that the two processes are interconnected. Modulating host cell death is critical for establishing bacterial attachment to the host and subsequent dissemination. Therefore, exploring the modes of LE involvement in host cell death is crucial for elucidating the mechanisms underlying EPEC infection and disease.

## INTRODUCTION

Enteropathogenic and enterohemorrhagic *Escherichia coli* (EPEC and EHEC) are extracellular diarrheagenic human pathogens contributing to significant morbidity and mortality worldwide (1, 2). Currently, only a few treatments are available. Therefore, a deep understanding of the molecular mechanisms by which these bacteria cause disease is indispensable for developing new therapeutics. EPEC and EHEC utilize a molecular syringe called the type III secretion system (T3SS) to deliver a set of “effector proteins” into the host cell (3). The type III secreted effectors contribute significantly to bacterial virulence because once within the host cell, these effectors can hijack and manipulate numerous host cell organelles and function in a way that contributes to successful bacterial colonization of the intestinal mucosa. Once attached to the apical surface of the intestinal epithelial cells, EPEC, EHEC, and the murine pathogen *Citrobacter rodentium* form a typical histopathological lesion in the mucosal surface of the gastrointestinal tract known as “attaching and effacing” (A/E). A/E is characterized by intimate microbial attachment to the apical cell plasma membrane of the enterocytes, local elimination of brush border microvilli, and the formation of actin-rich pedestal-like structures on top of which the bacterium resides (4–6).

The EPEC strain E2348/69 utilizes the T3SS to translocate into the host cell 21 effector proteins encoded by the locus of enterocyte effacement (LEE) and non-LEE genes (7). The first translocated effector is Tir (translocated intimin receptor) (8). Once translocated, Tir is incorporated into the host cell plasma membrane, exposing its extracellular domain for binding the bacterial outer membrane protein, intimin. Tir-intimin interactions are essential for promoting the firm attachment of the bacterium to its host cell and for pedestal formation (9, 10). Other injected effectors impact diverse processes of the host cell. For instance, Map is a guanine nucleotide exchange factor (GEF) for the Rho GTPase, Cdc42 (11–13). Map has also been shown to target host mitochondria (14–18), induce mitochondrial cell death (17, 18), promote endocytic turnover (19), mitogen-activated protein kinase (MAPK)/extracellular signal-regulated kinase (Erk)/MEK1/p38 signaling (17), and disruption of epithelial tight junctions (20). EspH is an actin cytoskeleton modulator that inhibits Rho GTPases (13, 21–23), host cell cytotoxicity (21), pedestal formation (24), and MAPK/Erk signaling (25). EspF, another mitochondrion-targeted effector (18, 26), facilitates host cell death (18, 27), endocytic trafficking at infection sites (19), and the disruption of epithelial tight junction barrier functions (28). NleB, NleH, NleF, and EspZ have been implicated in protecting against host cell death (18, 29–36). NleA inhibits the secretory pathway and disrupts epithelial barrier properties (37–39). EspG has been shown to regulate recycling endosomes (40, 41), inhibit the secretory pathway (42), microtubule dynamics (43, 44), actin cytoskeleton (45), and epithelial barrier functions (43, 46, 47). NleB, NleE, NleC, and NleD interfere with inflammatory signaling (48).

Lysosomes play a central role in trafficking from degradative endocytosis and are crucial for organismal homeostasis (49). Lysosomes can also act in autophagy (50), phagocytosis (51), and serve as Ca^+2^ and other ions storage organelles that control cell signaling (50, 52), metabolism (53, 54), and death (50, 55–58). One unique and well-studied event is the Ca^2+^-triggered lysosomal exocytosis (LE). This type of membrane trafficking response has been shown to play a pivotal role in repairing an injured plasma membrane (59–70) and be hijacked by several microbial pathogens. The best-studied pathogen in this respect is the parasite *Trypanosoma cruzi*, which induces LE and subsequent plasma membrane repair to promote its internalization into human host cells (71, 72). Adenoviruses adopt a similar strategy to infect host cells (73). LE linked to Ca^2+^ influx-dependent repair of injured membranes has been reported for the bacterial pore-forming toxins, streptolysin O from *Streptococcus* (59) and listeriolysin O from *Listeria monocytogenes* (74). LE has also been shown to be involved in *Salmonella enterica* (75), *Mycobacterium tuberculosis* (76), and *Helicobacter pylori* (77) pathogenesis, contributing to host cell killing or survival. However, despite this knowledge, there still needs to be a comprehensive mechanistic view of how pathogenic bacteria subvert LE to promote infection. Here, we identified three host cell death-modulating effectors, EspF, Map, and EspZ, of EPEC to be involved in controlling LE. The significance of these processes for EPEC infection is discussed.

## RESULTS

### EPEC stimulates lysosomal enzyme secretion

**Fig 1:**
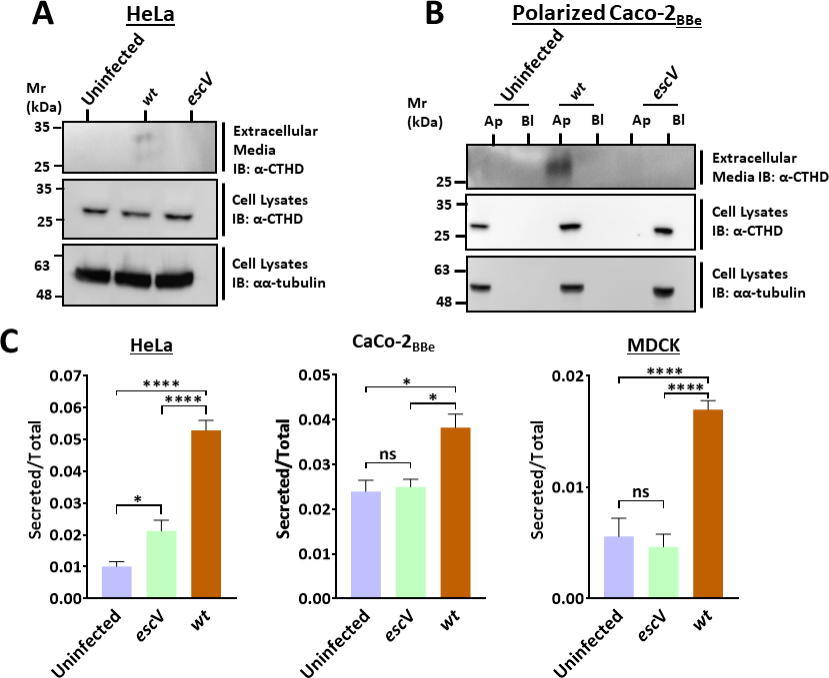
EPEC infection stimulates the secretion of lysosomal enzymes into the extracellular medium. (**A**) Secretion of CTHD from HeLa cells. HeLa cells were infected with EPEC-*wt* and EPEC-*escV* or remained uninfected. CTHD was detected in the extracellular media and cell lysates by immunoblotting (IB) using anti-CTHD antibodies. Probing with anti-α-tubulin antibodies was used to evaluate cell protein loading. A representative gel out of three independent experiments is shown. (**B**) Secretion of CTHD from polarized Caco-2_BBe_ cells. Polarized Caco-2_BBe_ cell monolayers were infected with the indicated EPEC strains or left uninfected. Media bathing the apical and basolateral surface of the cells were collected, and cells on filters were lysed, as described in Materials and Methods. CTHD and α-tubulin were detected in media and cell lysates by Western blotting. A representative gel out of three independent experiments is shown. (**C**) β-Hexosaminidase secretion. HeLa, Caco-2_BBe_, or MDCK cells were infected with the indicated EPEC strains or left uninfected. The extracellular media and cells were subjected to the β-hexosaminidase secretion assay described in Materials and Methods. Results are mean ± SE from four to six independent experiments.

To explore whether EPEC infection manipulates the secretion of lysosomal enzymes, HeLa or polarized Caco-2_BBe_ cells were infected with EPEC-*wt*, with the T3SS-deficient mutant EPEC-*escV*, or left uninfected. The presence of the lysosomal enzyme cathepsin D (CTHD) in the medium bathing the cells was examined by Western blotting. The results show that mature (~28 kDa) CTHD was present in media collected from EPEC-*wt*-infected HeLa cells (Fig. 1A) and in the medium contacting the apical but not the basolateral surface of the polarized Caco-2_BBe_ cells (Fig. 1B). Similarly, the activity of another lysosomal enzyme, β-hexosaminidase, in the extracellular medium of EPEC-*wt*-infected HeLa, Caco-2_BBe_, or MDCK cells was higher in the EPEC-*wt* compared to EPEC-*escV*, or uninfected cells (Fig. 1C). Collectively, these results suggest that EPEC stimulates type III secretion-dependent excretion of lysosomal enzymes into the extracellular milieu.

### EspF and Map drive lysosomal enzyme secretion

**Fig 2:**
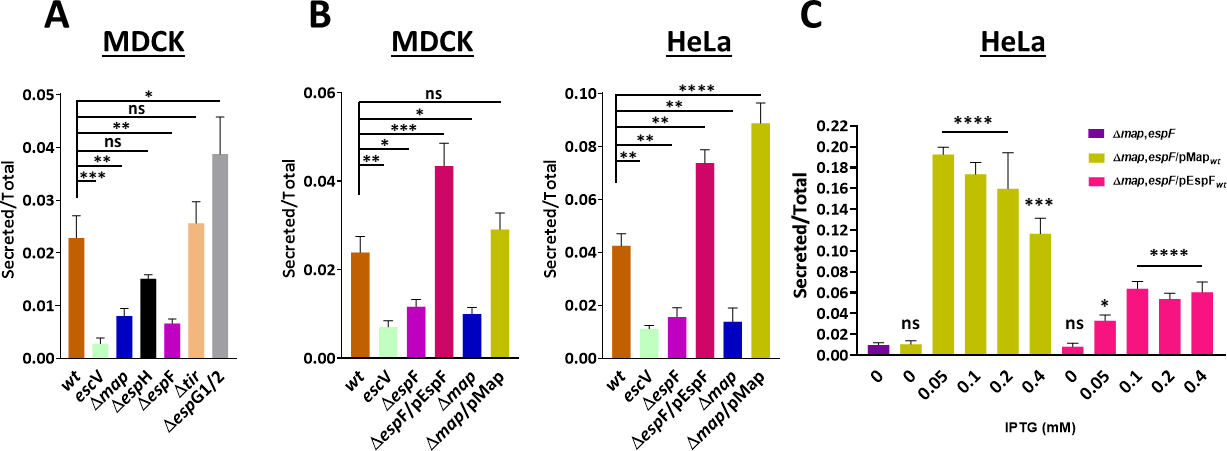
Identifying type III secreted effectors that exert β-hexosaminidase secretion. (**A**) Screening for LEE-encoded effectors. MDCK cells were infected with the indicated EPEC strains, and the β-hexosaminidase secretion assay was applied, as described in Materials and Methods. Results are mean ± SE from seven independent experiments. (**B**) Effects of EspF and Map. MDCK or HeLa cells were infected with the indicated EPEC strains, and the β-hexosaminidase release assay was performed. Results are mean ± SE from 10 (MDCK) and 4 (HeLa) independent experiments. (**C**) The relative contribution of EspF and Map. HeLa cells were infected for 60 min at 37°C with the indicated EPEC strains. The indicated isopropyl-β-D-thiogalactopyranoside (IPTG) concentrations have been used to induce the effector expression, and the cells were subjected to the β-hexosaminidase release assay. Results are mean ± SE of three independent experiments. Asterisks indicate statistical significance compared to ∆*map, espF*.

Our next aim was to identify type III secreted effectors encoded by LEE genes that prompt lysosomal enzyme secretion. For this, MDCK cells were infected with EPEC-Δ*map*, EPEC-Δ*espH*, EPEC-Δ*espF*, EPEC-Δ*tir,* and EPEC-Δ*espG1/2* mutant strains, and the β-hexosaminidase activity assay was applied to the extracellular medium of the infected cells, as before. In these experiments, infection with EPEC-*wt* and EPEC-*escV* was positive and negative controls, respectively. Compared to EPEC-*wt*, only infection with EPEC-Δ*espF*, or EPEC-Δ*map*, resulted in a significant reduction in β-hexosaminidase secretion (Fig. 2A), suggesting that the two effectors play a role in mediating EPEC-dependent lysosomal enzyme secretion.

To investigate this further, MDCK or HeLa cells were infected with the EPEC-Δ*espF*, or EPEC-Δ*map*, or with EPEC mutants complemented with plasmids encoding for the respective bacterial effectors, i.e., EPEC-Δ*espF*/pEspF or EPEC-Δ*map*/pMap. As before, infection with EPEC-*wt* and EPEC-*escV* was positive and negative controls, respectively. The results clearly show that infection with the mutant EPEC strains caused reduced β-hexosaminidase activity in the medium bathing the cells. However, infection with the complemented bacterial strains has rescued the effect (Fig. 2B).

Notably, the translocated EspF and Map effectors have been shown to promote host cell death by targeting host mitochondria (17, 18, 27, 78). Lytic cell death was evaluated using the lactate dehydrogenase (LDH) release and propidium iodide (PI) uptake assays, which report plasma membrane rupture/permeability occurring during the induction of some of the programmed cell death pathways (79, 80). Indeed, while infection with the EPEC-Δ*espF* or EPEC-Δ*map* mutants resulted in low LDH release and PI uptake levels, infection with the EPEC-Δ*espF*/pEspF or EPEC-Δ*map*/pMap complemented strains increased these levels (Fig. S1).

In previous studies using an EPEC-Δ*map,espF* double mutant strain complemented with a plasmid encoding either Map (Δ*map,espF*/pMap) or EspF (Δ*map,espF*/pEspF), we showed that Map was more effective than EspF in targeting host mitochondria and triggering host cell apoptosis (17). Map and EspF’s relative contribution to LE stimulation was examined using a similar experimental approach. HeLa cells were infected with the indicated EPEC strains, and the level of β-hexosaminidase release to the extracellular medium was measured. Different isopropyl-β-D-thiogalactopyranoside (IPTG) concentrations were used to induce the effector expression. The results show that compared to HeLa cell infection with EPEC-Δ*map,espF*, infection with EPEC-Δ*map,espF*/pMap or Δ*map,espF*/pEspF stimulated LE (Fig. 2C), suggesting that Map and EspF can independently promote the process. However, in the absence of EspF, Map exerts a more potent effect (Fig. 2C), an effect that can be contributed by the more efficient trafficking of Map to mitochondria (17). One cannot exclude the existence of collaboration between the two effector proteins mediating the effect.

Altogether, these results suggest that EspF and Map drive lysosomal enzyme secretion and that the effect is not cell type specific. Additionally, this activity correlates with their capacity to induce lytic cell death, suggesting that the capabilities of EspF and Map to release lysosomal enzymes and cause cell death are somehow interconnected.

### The role of EspF and Map domains

**Fig 3:**
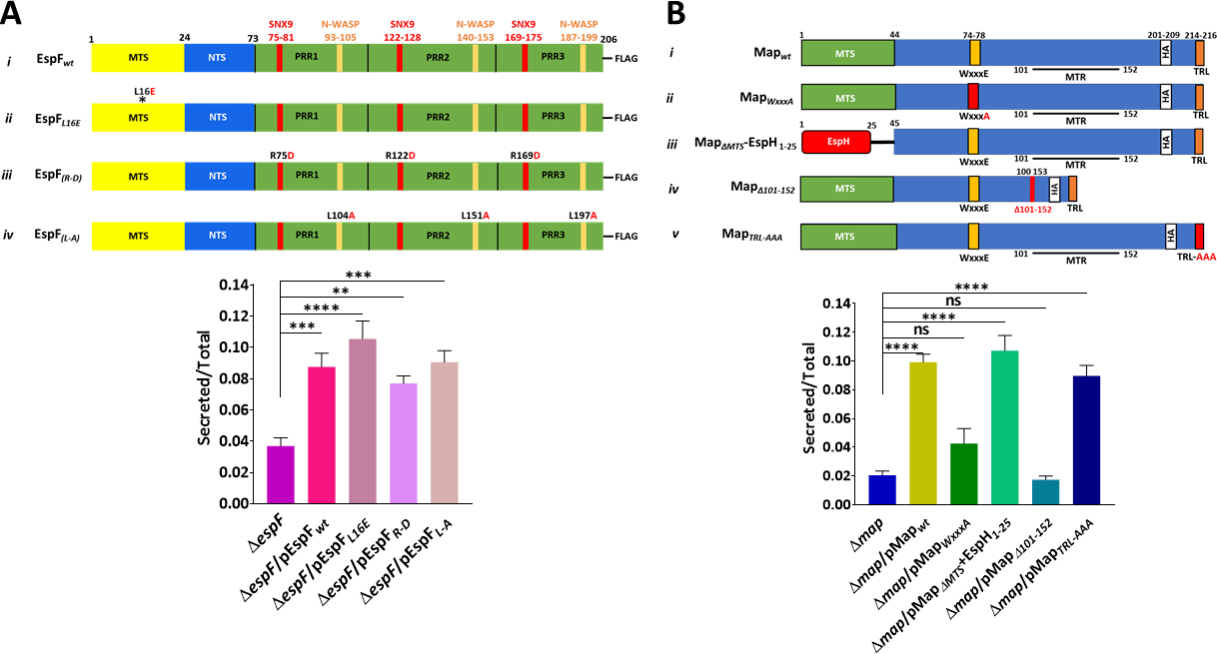
Effects of EspF and Map domains on lysosomal enzyme secretion. (**A**) Effects of EspF domains. Upper panel: Schematic of the C-terminally FLAG-tagged EspF_*wt*_ and EspF mutations used to inactivate each EspF domain. (i) The names of each EspF*_wt_* domain (MTS [26]; NTS [85]; SNX9 [86]; N-WASP [82]) are shown. The three redundant PRR domains of EspF are also indicated. The amino acids are numbered starting with 1 in the N-terminus. (ii) L16 is a leucine positioned in the N-terminal mitochondrial targeting sequence (marked with an asterisk). Its mutation to glutamate (L16E) abrogates mitochondrial targeting of EspF. (iii) The arginine-to-aspartic acid (R-D) mutations in each PRR inactivate SNX9 binding. (iv) The leucine-to-alanine (L-A) mutation in each PRR abolishes N-WASP binding. Lower panel*:* HeLa cells were infected with the indicated EPEC strains, and the β-hexosaminidase secretion assay was performed as described. Results are mean ± SE from five independent experiments. (**B**) Effects of Map domains. Upper panel: Schematic of the HA-tagged Map_*wt*_ effector protein and the mutations used to inactivate each Map domain. (i) The MTS (14, 17), the WxxxE RhoGEF (11), the mitochondrial toxicity region (MTR) (14), and the C-terminal TRL PDZ type I binding motif (87) are shown. The amino acids are numbered in sequence, starting with the N-terminus of the protein. (ii–v) The mutations used to inactivate each domain are indicated in red. (ii) The glutamate on the WxxxE GEF domain is mutated to alanine; (iii) the N-terminal MTS is replaced with the EspH N-terminal 25 amino acid; (iv) amino acids 101–152 of the MTR motif are removed; (v) the C-terminal TRL amino acids, which constitute the PDZ-type I binding motif, are substituted to alanine. All mutations have been described in reference (17). (**A**) Lower panel*:* HeLa cells were infected with the indicated EPEC strains, and the β-hexosaminidase secretion assay was applied. Results are mean ± SE from five independent experiments.

EspF and Map are multifunctional effectors with several domains mediating their interactions and functions within the host (reviewed in references 81–84). To gain a deeper insight into the mechanisms by which EspF and Map promote lysosomal enzyme secretion, the contribution of each of these domains in promoting β-hexosaminidase release has been examined. EspF harbors an N-terminal mitochondrial targeting sequence (MTS), which contains a critical leucine at position 16. It includes an additional nucleolar targeting signal (NTS), sorting nexin-9 (SNX9) and neuronal Wiskott-Aldrich syndrome protein (N-WASP) binding motifs, encoded by each of the three proline-rich regions (PRR) (illustrated in Fig. 3A, upper, i, reviewed in references 81, 83, 84). The motifs have been mutated (Fig. 3A, upper, ii*–*iv), and the EspF mutants were expressed in EPEC-Δ*espF* to generate the following complemented bacterial strains: EPEC-Δ*espF* + EspF*_L16E_* (Fig. 3A, upper, ii; deficient in mitochondrial targeting), EPEC-Δ*espF*/pEspF*_R-D_* (Fig. 3A, upper, iii; defective in SNX9 binding), and EPEC-Δ*espF*/pEspF*_L-A_* (Fig. 3A, upper, iv; deficient in N-WASP binding). HeLa cells were infected with these bacterial strains, and the β-hexosaminidase release assay was applied to measure the lysosomal secretion from the cells, compared to EPEC-Δ*espF*-infected cells. Consistent with data shown in Fig. 2B, cell infection with EPEC-Δ*espF*/pEspF enhanced β-hexosaminidase secretion from the cells. Infection with EPEC-Δ*espF* complemented with the indicated EspF mutant also showed induced β-hexosaminidase secretion to levels similar to those displayed by the EPEC-Δ*espF* + EspF*wt*-infected cells (Fig. 3A, lower). As the levels of mutant effector translocation were similar in all cases (Fig. S2A), these data suggest that EspF domains are not involved in the EspF-dependent secretion of β-hexosaminidase.

Map has also been characterized to contain several functional domains: an N-terminal MTS, a WxxxA Rho GEF motif, a mitochondrial toxicity region (MTR), and a C-terminal PDZ type I binding sequence, TRL (Fig. 3B, upper, i) (reviewed in reference 83). Each of these motifs has been mutated (Fig. 3B, upper, ii*–*v) and expressed in EPEC-Δ*map* to generate: EPEC-Δ*map*/pMap*_WxxxA_* (Fig. 3B, upper, ii; deficient in Rho GEF activity), EPEC-Δ*map*/pMap_Δ_*_MTS_*-EspH_1-25_ (Fig. 3B, upper, iii; deficient in mitochondrial targeting [17]), EPEC-Δ*map*/pMap_Δ_*_101-152_* (Fig. 3B, upper, iv; deficient in mitochondrial toxicity activity), or EPEC-Δ*map*/pMap*_TRL-AAA_* (Fig. 3B, upper, v; unable to interact with PDZ type I sequences [88]).

Consistent with data presented in Fig. 2B, HeLa cell infection with EPEC-Δ*map*/pMap*_wt_* resulted in an augmented β-hexosaminidase secretion compared to EPEC-Δ*map*. While infection with EPEC-Δ*map*/pMap*_ΔMTS_*-EspH_1-25_ or EPEC-Δ*map*/pMap*_TRL-AAA_* has also stimulated lysosomal enzyme secretion, infection with EPEC-Δ*map*/pMap*_WxxxA_* or EPEC-Δ*map*/pMap*_Δ101-152_* caused a significant reduction in β-hexosaminidase release, reaching levels exhibited by EPEC-Δ*map*-infected cells (Fig. 3B, lower). Map was translocated into the host cells at roughly comparable levels, except for Map_TRL-AAA_, whose translocation level was higher (Fig. S2B). Bacterial exposure to increasing IPTG concentrations did not significantly affect the β-hexosaminidase release levels in EPEC-map/pMap101-152-infected cells (Fig. S3A). In contrast, in EPEC-Δ*map*/pMap_WxxxA_-infected cells, the lysosomal enzyme secretion levels have somewhat increased in response to elevated IPTG levels yet not reaching the levels exhibited by EPEC-Δ*map*/pMap-infected cells (Fig. S3B). These data suggest that mutations in Map’s WxxxE GEF and MTR domains certainly impacted its capacity to induce lysosomal enzyme secretion, thus highlighting their involvement in mediating Map-dependent lysosomal enzyme secretion. Finally, a complete correlation between the ability of the effector mutants to induce β-hexosaminidase secretion (Fig. 2 and 3) and lytic cell death measured by LDH release (Fig. S1) was observed, suggesting again that induced lysosomal enzyme release and lytic cell death are interlinked processes.

### The pro-death effectors EspF and Map promote the concomitant appearance of Lamp-1 on the infected host cell surface and lytic cell death

**Fig 4:**
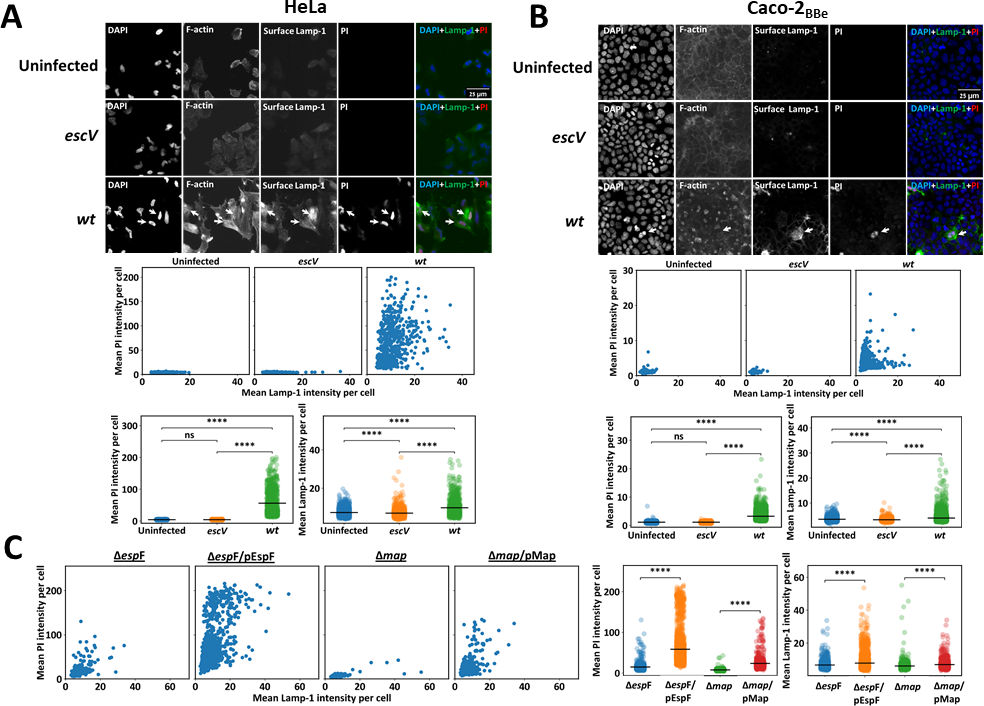
Effects of EPEC infection on PI uptake and Lamp-1 surface appearance in single cells. (**A**) Effects of EPEC-*wt* and EPEC-*escV* infection on HeLa cells. HeLa cells were infected with pre-activated EPEC-*escV*, or EPEC-*wt*, or remained uninfected. Cells were exposed to PI, and their surface was immunostained with anti-Lamp-1 antibodies recognizing the protein’s extracellular domain. Cells were then fixed, permeabilized, stained with DAPI and phalloidin-CF647, and processed for fluorescence microscopy. Representative images from three independent experiments are shown (upper panel). The mean PI vs Lamp-1 intensities per cell were determined and plotted on scatter and strip plots (lower panels). The scatter plots combine data from four different optical fields of view taken from two independent experiments, encompassing 1,000–1,500 cells in each condition (Control, *escV*, or *wt*). For each condition, Lamp-1 and PI fluorescence mean intensities were also plotted in a strip plot (lower right panels), whereby the black lines in the strip plots represent the mean intensity. A two-sided Welch’s *t*-test and Bonferroni’s correction were used to determine statistical significance. ****P = 1e−4, ns = not-significant. (**B**) Effects of EPEC-*wt* and EPEC-*escV* infection on Caco-2_BBe_ cells. Cells were seeded on collagenated coverslips, infected with pre-activated EPEC-*escV* or EPEC-*wt*, exposed to PI uptake, immunostained with anti-Lamp-1 antibodies under conditions that allowed surface labeling, stained with DAPI and phalloidin-CF647, and processed for fluorescence microscopy, as in panel A. Representative images are shown (upper panel). The mean PI vs Lamp-1 intensities per cell were determined and plotted on scatter and strip plots (lower panels). A two-sided Welch’s *t*-test and Bonferroni’s correction were used to determine statistical significance. ****P = 1e−4, ns = not-significant. (**C**). Effects of EspF and Map. HeLa cells were infected with the indicated EPEC strains, and the PI and surface Lamp-1 fluorescence levels were analyzed, as described above and in Materials and Methods. A two-sided Welch’s *t*-test was applied to determine statistical significance. ****P = 1e−4, ns = not-significant.

We developed a fluorescence microscopy-based single-cell analysis of these processes to examine further the tight linkage between the induction of LE and lytic cell death. The stimulated release of lysosomal enzymes into the extracellular environment results from the fusion of the lysosomal limiting membrane and the plasma membrane, resulting in the appearance of lysosomal membrane proteins, e.g., Lamp-1, on the infected cell’s plasma membrane (89). If lytic cell death and LE are related processes, one would expect that individual infected cells will show a simultaneous appearance of Lamp-1 on their cell surface (“Surface Lamp-1”), and permeability to PI added to the extracellular medium. To address this prediction, HeLa or Caco-2_BBe_ cells were infected with EPEC-*escV*, EPEC-*wt,* or left uninfected. Then, the cells were exposed to PI in the cold and to anti-Lamp-1 antibodies, recognizing specifically the ectopic portion of the lysosomal membrane protein. Cells were then fixed and immunostained with fluorescent secondary antibodies, permeabilized, and stained with Phalloidin-CF647 to label F-actin and DAPI to visualize bacterial and host DNA. The mean PI and surface Lamp-1 intensities per cell were quantified, as described in Materials and Methods. The results show a substantial and concomitant increase in the mean PI and Lamp-1 surface intensities per cell in EPEC-*wt*, compared to EPEC-*escV* and uninfected cells (Fig. 4A and B). A similar increase was also observed in EPEC-Δ*espF*/pEspF- or EPEC-Δ*map*/pMap-infected cells compared to their respective EPEC-Δ*espF* and EPEC-Δ*map* mutant strains (Fig. 4C). These data strengthen the conclusion that EPEC-induced LE and lytic cell death are linked processes.

### The anti-death effector, EspZ, inhibits LE and lytic cell death

**Fig 5:**
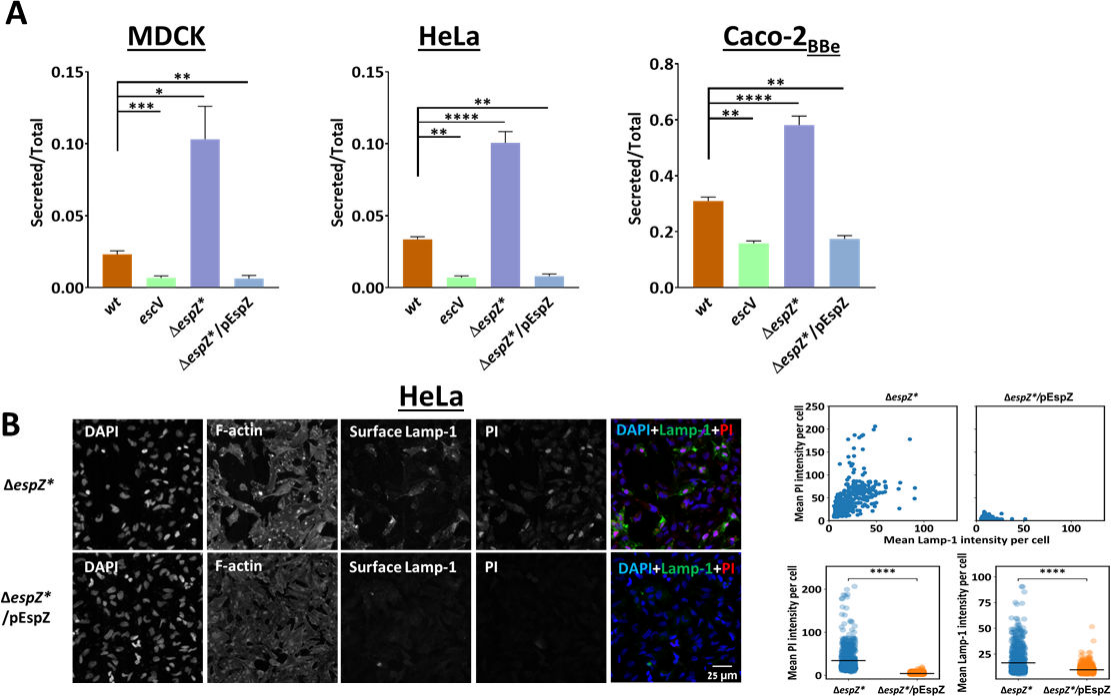
Effects of EspZ on β-hexosaminidase release, cell permeability to PI, and Lamp-1 surface expression. (**A**) Effects on β-hexosaminidase release. MDCK, HeLa, or Caco-2*_BBe_* cell monolayers were infected with EPEC-*escV*, EPEC-*wt*, EPEC-ΔespZ*, and EPEC-ΔespZ*/pEspZ, and the β-hexosaminidase release was applied, as before. Results are mean ± SE from four independent experiments. (**B**) Effects on PI uptake and Lamp-1 surface expression in single cells. HeLa cells were infected with the indicated EPEC strains, and the PI and surface Lamp-1 fluorescence levels were analyzed as in Fig. 4. A representative fluorescence microscopy image is shown (left panel), and the mean fluorescence intensities of PI and Lamp-1 are shown as scatter and strip plots (right panels). A two-sided Welch’s *t*-test was performed to determine the statistical significance. ****P = 1e−4, ns = not-significant.

SepZ/EspZ is an essential type III secreted effector (90, 91) that protects against host cell death induced by EPEC-Δ*espZ* infection (29, 36, 92, 93). Given the correlative effects on host cell death and LE, infection with EPEC-ΔespZ* is expected to induce LE and lytic cell death, while infection with EPEC-ΔespZ*/pEspZ is not. To address this prediction, an EPEC-ΔespZ* strain expressing EspZ has been generated (EPEC-ΔespZ*/pEspZ; see Materials and Methods, Table S1, and schematic depiction of the EspZ construct in Fig. S4A). The T3SS-dependent translocation of EspZ (Fig. S4B and C) and protection against lytic cell death conferred by EPEC-Δ*espZ* (Fig. S4D) have been confirmed in HeLa-infected cells (see also reference 36). A significant increase in β-hexosaminidase release from MDCK, HeLa, or Caco-2_BBe_ cells was observed upon infection with EPEC-ΔespZ* (Fig. 5A). In contrast, cell infection with EPEC-ΔespZ*/pEspZ imposed a substantial inhibition of β-hexosaminidase release, reaching levels displayed by EPEC-*escV*-infected cells (Fig. 5A). Additionally, while infection with EPEC-ΔespZ* caused a concomitant increase in PI uptake and Lamp-1 surface expression per cell, infection with EPEC-ΔespZ*/pEspZ resulted in significantly lower levels of the measured parameters (Fig. 5B). These results further emphasize the link between LE and cell death during EPEC infection.

### Tir induces clustering of surface Lamp-1 at infection sites

**Fig 6:**
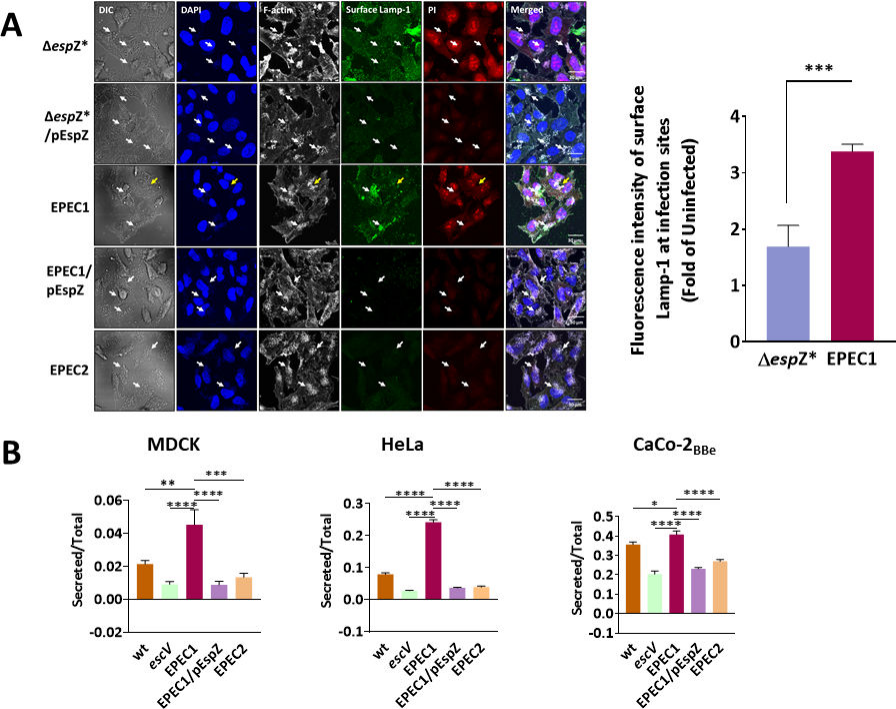
EPEC1 and EPEC1 expressing EspZ (EPEC1/pEspZ and EPEC2) effects on surface Lamp-1 clustering at infection sites and lysosomal enzyme secretion. (**A**) Impacts on surface Lamp-1 clustering. HeLa cells were infected with the indicated EPEC strains. Cells were subjected to PI uptake and Lamp-1 surface immunostaining, fixed, permeabilized, and stained with DAPI (to visualize host cell nuclei and bacterial microcolonies) and TR-phalloidin (to visualize F-actin). Cells were then imaged with confocal microscopy. Representative images of three independent experiments are shown (A, left). Arrows point toward infection sites. In the case of EPEC1 infection, white arrows indicate infecting microcolonies in which surface Lamp-1 has been visualized at the injection site, and yellow arrows point to a microcolony in which surface Lamp-1 clustering has not been detected. The degree of Lamp-1 clustering has been quantified, as previously described (19) (A, right). (**B**) Effects on lysosomal enzyme secretion. MDCK, HeLa, and Caco-2_BBe_ cells were infected with the indicated EPEC strains, and β-hexosaminidase secretion levels were determined, as described in Fig. 1C and Materials and Methods. Results are mean ± SE from *n* = 4 to 6 independent experiments.

In EPEC-Δ*esp*Z*-infected cells, the surface Lamp-1 staining is spread over the entire infected cell surface, with minor accumulation at infection sites. As expected, slight surface Lamp-1 staining has been observed in EPEC-Δ*espZ*/pEspZ-infected cells (Fig. 6A). Host cell nuclei were prominently stained with PI, suggesting that the plasma membrane of these cells became permeable to the dye, likely due to the induction of lytic cell death. Studies have shown that infection with EPEC1, an EPEC strain that expresses only Tir, induces inflammatory lytic cell death (pyroptosis) (92, 94). Tir bound to intimin is confined to plasma membrane infection sites. Studies have also shown that pyroptotic cell death induced by EPEC1 is initiated by Ca^2+^ influx and signaling at these sites (94). Given that LE is triggered by Ca^2+^ (60), we reasoned that the fusion of lysosomes with the host cell plasma membrane in EPEC1-infected cells would be limited to infection sites, thereby restricting the distribution of surface Lamp-1 to those sites.

EPEC1 stimulates lysosomal enzyme secretion in MDCK, HeLa, and Caco-2_BBe_ cells. Infection with EPEC1/pEspZ, or EPEC2, which expresses endogenous Tir and EspZ only (83, 89), suppresses this effect (Fig. 6B). Interestingly, in agreement with our hypothesis, clusters of surface-stained Lamp-1 have been observed at EPEC1 infection sites in HeLa cells (Fig. 6A; Fig. S5). The nuclei of the infected cells were also stained with PI (Fig. 6A; Fig. S5), consistent with the ability of Tir to evoke lytic cell death. Infection with either EPEC1/pEspZ, or EPEC2, resulted in a significant loss in surface Lamp-1 and PI nuclei staining (Fig. 6A), suggesting that translocated EspZ inhibits the EPEC1-evoked LE and lytic cell death. Together, these results demonstrate that LE occurs in response to cell death triggered by EPEC lacking EspZ or by EPEC expressing Tir only and that translocated EspZ antagonizes the two pro-death evoked processes.

## DISCUSSION

Our findings show that EPEC induces host cell death and LE simultaneously, suggesting the two processes are somehow interconnected. What is the reason for the tight linkage? One possibility is that LE is a host cell response to the induction of cell death. For instance, EspF and Map trigger a mitochondrial cell death program, which causes plasma membrane permeability (18). The host cell reacts to this process by eliciting LE to repair the damaged plasma membrane (see below). Lysosomes have been implicated in triggering programmed cell death, mainly when their limiting membrane is compromised and becomes permeable (55–58, 95–97). Hence, another scenario is that the bacterial effectors target lysosomes in a way that damages their limiting membrane, triggering cell death and LE. This “damage response” may help the infected cell to clear itself of damaged lysosomes. Similar results can be achieved by lysophagy (98). In this context are studies showing the existence of physical and functional interactions between mitochondria and lysosomes (99–101). Interestingly, data have shown that mitochondrial dysfunction can impair the structure and function of lysosomes and that lysosomal impairment depends on reactive oxygen species [reviewed in reference (102)]. Studies have also shown that damaged lysosomes can affect mitochondrial functions (103). To our knowledge, EPEC effectors targeting lysosomes have not yet been identified. It would be, hence, interesting to uncover the modes by which EPEC effectors target these interconnected organelles to promote LE and host cell death.

LE and lytic host cell death are mediated by at least three cell death modulating effectors: the pro-death EspF and Map and the anti-death, EspZ. Our data also indicate that mitochondrial targeting of EspF and Map is not involved in mediating these effects because mutational inactivation of the MTS did not affect LDH release and LE (Fig. 3B; Fig. S1). However, Map-induced host cell death and LE can be exerted by altering mitochondrial morphology, as residues 101–152 of the Map MTR have been identified to play a role in this process (Fig. 3B; Fig. S1) (14, 17). Although the mechanism by which Map-mediated alterations in mitochondrial morphology lead to mitochondrial dysfunction and lytic cell death is not known, it is interesting to highlight recent findings linking mitochondria, reactive oxygen species production, and inflammatory cell death (104). The failure of the EPEC-Δ*map* + Map*_WxxxA_* to induce lysosomal enzyme secretion and cell death (Fig. 3B; Fig. S1) suggests that Cdc42 activation by Map plays a role in these processes. These results are consistent with studies suggesting that manipulating RhoGTPase activity by the effector YopE can regulate cell death in *Yersinia enterocolitica*-infected (105) and healthy cells (106).

Lysosomal exocytosis is driven by Ca^2+^ influx (107). The clustering of host-translocated Tir during EPEC infection triggers Ca^2+^ influx at infection plasma membrane sites and pyroptosis of the infected intestinal cells (92). Hence, the clustering at infection sites of surface Lamp-1 in EPEC1 (expressing Tir only)-infected cells (exemplified in Fig. 6A ; Fig. S6) could be attributed to local Tir-triggered Ca^2+^ influx, which induces localized fusion of lysosomes with the infected plasma membrane, resulting in preferential insertion and localization of Lamp-1 at the infection sites. Such a phenomenon was vaguely seen in EPEC-ΔespZ*-infected cells (Fig. 6A). This could be attributed to the translocation of other pro-death effectors (e.g., EspF and Map), which unlike Tir, cause lytic cell death by impacting internal organelles (e.g., mitochondria) of the EPEC-ΔespZ*-infected cells. Map, for instance, has been shown to produce a Ca^2+^ wave that propagates throughout the entire cell volume (17). Such a wave may promote unrestricted lysosomal membrane-plasma membrane fusion and Lamp-1 appearance on the infected cell surface.

What is the functional significance of LE and lytic host cell death in the host and bacterial lifestyle? Ample studies suggest that Ca^2+^-regulated LE represents a cell response that enables a rapid healing (repair) of a wounded (damaged) plasma membrane. Mechanistically, this could be achieved by plasma membrane exposure to lysosomal acid sphingomyelinases, resulting in the generation of tightly packed ceramide platforms in the outer leaflet of the cell membrane, facilitating endocytic removal of the damaged plasma membrane. Combined with exocytosis and endocytic recycling of membranes, which enrich the plasma membrane with proteins and lipids, these processes repair plasma membrane damage (60, 61, 63, 108–111). In this context, it is worth pointing out our recently published findings suggesting that EspF and Map facilitate endocytic turnover at EPEC infection sites (19). Efficient plasma membrane repair at the onset of infection would keep the host cell alive, which is essential to allow bacterial attachment to the host surface and productive colonization. An opposing scenario could link LE to bacterial spread. Studies have shown that the induction of sphingomyelinases and ceramide production can trigger cell death (112, 113). Therefore, LE and exposure to lysosomal acid sphingomyelinases could facilitate host cell death, bacterial dissemination, and replication in uninfected tissues. Data potentially supporting this hypothesis suggest that membrane remodeling in response to acid sphingomyelinase activity is involved in *Shigella flexneri* replication within epithelial hosts (114). The role of LE and host cell death in A/E bacterial pathogen infection needs further exploration.

## MATERIALS AND METHODS

### Cell culture

HeLa, MDCK II, and Caco-2_BBe_ (polarized or semi-polarized) cells were cultured, as before (17, 19, 25, 115, 116). All tissue culture media and buffers were from Biological Industries.

### Cloning of EspF, Map, and EspZ into the pSA10 bacterial expression vector

The standard Gibson assembly method (117) and the Gibson assembly master mix (NEB E2611) were used to clone the bacterial genes into the pSA10 vector, according to the manufacturer’s instructions. Nucleotide sequences of all constructs were confirmed by the Genomic Technologies Facility (https://www.bio.huji.ac.il/en/units_the_national_center_for_genomic_technologies) using Sanger sequencing.

#### Construction of pSA10-EspF-FLAG and its mutants

pSA10-EspF-FLAG was constructed by replacing the EspH-6×His-streptavidin-binding peptide (SBP) fragment of the pAA6284 plasmid vector (Table S2) with EspF-FLAG. The EspF-FLAG insert was PCR amplified using 1F′ and 2R′ primers (Table S3) from a pJN61-EspF-FLAG plasmid (Table S2). The pAA6284 vector was linearized by PCR using the primers 3F′ and 4R′ (Table S5). EspF mutants were constructed on pSA10-EspF-FLAG, as follows. Oligonucleotides 5F′ and 6R′ (Table S3) were used to mutate leucine (L) at position 16 to glutamic acid (E) and generate the pSA10-EspF_L16E_ plasmid (Table S2). The pSA10-EspF_R-D_ plasmid (Table S4) was constructed by linearizing pSA10-EspF-FLAG using the 8F′ and 9R′ oligonucleotides (Table S3) and inserting the gblock7 (Table S3). In this mutant, arginine (R) in positions 75, 122, and 169 was mutated to aspartic acid (D). The pSA10-EspF_L-A_ plasmid (Table S2) was constructed by linearizing pSA10-EspF-FLAG using 11F′ and 12R′ (Table S3) and inserting the gblock10 (Table S3). In this mutant, leucine (L) in positions 104, 151, and 197 was mutated to alanine.

#### Construction of pSA10-Map_TRL-AAA_ mutant

All Map constructs contain a hemagglutinin (HA)-tag (YPYDVPDYA) insert at positions 201–209. pSA10-Map_TRL-AAA_ was constructed on the pSA10-Map plasmid (Table S2) by mutating the C-terminal TRL (214–216) to alanine AAA. Substitution of the conserved C-terminal PDZ class I binding sequences to alanine abolishes their interaction with PDZ domains (88). The vector was linearized using the 13F′ and 14R′ oligonucleotides (Table S3), and the insert was mutated by PCR amplification using oligonucleotides 15F′ and 16R′ (Table S3).

#### Construction of pSA10-EspZ-2×HA-SBP

The construction of the plasmid has been recently described (36). Briefly, pSA10-EspZ-2xHA-SBP plasmid was constructed by replacing EspH-6×His-SBP of pAA6284 (Table S2) with EspZ-2×HA. Oligonucleotides 17F′ and 18R′ (Table S3) were used to obtain the PCR-linearized pAA6284 vector, and oligonucleotides 19F′ and 20R′ (Table S3) were used to PCR amplify EspZ from the mCherry-EspZ template (Table S2). Oligonucleotides 21F′ and 22R′ (Table S3) were used to PCR amplify 2×HA from the gblock23 template (Table S3).

### Bacterial strains

Bacterial strains are listed in Table S1. Mutants were manufactured using the lambda red method (118). Notably, the EPEC-ΔespZ* mutant strain was made in the background of the IE6- and PP4-deleted islands. These two genomic islands encode effectors counteracting host cell death (e.g., *nleB*, *nled*, and *nleH*; reviewed in reference 119). Thus, deleting these genes is expected to enable the examination of the net-maximal effect that the *espZ* deletion has in promoting host cell death.

### Plasmid electroporation into EPEC mutant strains

Effector-encoding plasmids were electroporated at 1.85 kV/25 µF/200 Ohm, using the BioRad electroporator (Gene Pulser II) into the respective EPEC mutant strains to generate the complemented EPEC strains (Table S1).

### SDS-PAGE and Western blotting

SDS-PAGE and Western blotting were performed, as described (17). Briefly, samples were lysed with sample buffer (40% glycerol, 12% SDS, 0.2M Tris-HCl pH-6.8, and 100 mM dithiothreitol) supplemented with bromophenol blue. The lysate was then heated at 95°C for 10 min, and the proteins were separated by SDS-PAGE (BioRad Mini-PROTEAN Tetra system; 40 mA, 30 min), transferred to nitrocellulose membrane (Bio-Rad Trans-Blot Turbo; 2.5 A, 10 min), and blocked with TBST + 2.5% BSA + 1% milk. The membranes were probed with respective antibodies diluted in TBST + 5% BSA for 1 h at 22°C or overnight at 4°C with gentle shaking. The membranes were imaged using a Fusion FX spectra imager (Vilber Smart Imaging, Collègien, France), and the band intensity was measured using Fiji (NIH).

### Bacterial activation and cell infection

The T3SS of all bacterial strains used in this study was activated for 3 h at 37°C and 5% CO_2_ before infection, as described in reference 17. In EPEC strains complemented with Map, EspF, and EspZ plasmids-infected cells, the protein expression was induced by supplementing the activation medium with isopropyl-β-D-thiogalactopyranoside (Promega, Madison, WI) during the last 30 min of activation as follows: Δ*map* + Map*_wt_*, 0.1 mM; Δ*map* + Map*_wxxxA_*, 0.2 mM; Δ*map* + Map*_∆MTS_*, EspH_1-25_, 0.2 mM; Δ*map* + Map*_Δ101-152_*, 0.05 mM; Δ*map* + Map*_TRL_*, 0.2 mM; Δ*esp*F + EspF*_wt_*, 0.1 mM; Δ*esp*F + EspF*_L_*, 0.05 mM; Δ*esp*F + EspF*_R_* , 0.4 mM; Δ*esp*F + EspF*_L_* , 0.4 mM; Δ*esp*Z* + EspZ-2×HA-SBP, 0.2 mM. Under these IPTG concentrations, infection with the EPEC-Δ*espF* and Δ*map* mutants yielded maximal β-hexosaminidase release compared to EPEC-Δ*espF* + EspF and EPEC-Δ*map* + Map*_wt_*, respectively. Infections were performed in a CO_2_ incubator (37°C, 5% CO_2_, 90% humidity) for 1 h (HeLa and CaCo-2_BBe_) and 2 h (MDCK) cells, as described (17).

### Lactate dehydrogenase cytotoxicity assay

HeLa cells (50,000 cells/well) were seeded on a 24-well plate and incubated for 48 h in a CO_2_ incubator (37°C, 5% CO_2_, 90% humidity). Cells were then infected with pre-activated EPEC strains for 1 h at 37°C. Media bathing the cells were collected and centrifuged (600 g, 10 min), and the LDH released into them was measured by the LDH-cytotoxicity assay (Abcam #ab65393), as described in the manufacturer’s protocol. The percentage LDH release (i.e., cytotoxicity [%]) = $\frac{Test\;Sample-Low\;control}{High\;control-Low\;control}\times 100$, whereby the Test Sample, infected cells; Low control, uninfected cells; High control, Low control + lysis solution.

### Propidium iodide uptake assay

The PI uptake assay was applied as described (92), with some modifications. HeLa cells (5 × 10^4^ cells/well) were seeded in a black clear-bottom 96-well plate (Greiner Bio-One, 655090) 1 day before infection. Cells were washed and incubated with phenol-red-free DMEM for 15 min before infection. Bacteria were primed in phenol-red-free DMEM (high glucose; Biological Industries, 01-053-1A) for 3 h. PI (5 µg/mL; Sigma, P4170) was added to the activated bacteria. The cell medium was replaced with the PI-containing activated bacteria. The PI fluorescence was measured after 150 min incubation (37°C; 5% CO_2_ BioTek Synergy H1 plate reader; 520 nm excitation and 620 nm emission wavelengths). PI-containing plain media served as blanks. Cells not exposed to the bacteria but otherwise treated equally were designated as “uninfected.” Positive controls were cells solubilized in 0.1% Triton X-100 (JT Baker; X198-07) and treated with PI. These cells were immediately subjected to fluorescence measurements taken in 30-min intervals. Plateau levels were averaged and used as the “positive control” values. Following subtraction of the blank from each reading, PI uptake percent was calculated as follows: $\frac{Infected-Uninfected}{Positive\;control}\times 100$.

### Cathepsin D detection

HeLa cells were seeded on a 6-well plate (~160,000 cells/mL) and grown for 2 days until reaching ~70% confluence. Caco-2_BBe_ (625,000 cells/mL) were seeded on collagenated 24-mm Transwells under conditions that allowed them to form polarized cell monolayers, as described (116). Cells were infected with pre-activated EPEC for 60 min (for HeLa) and 120 min (for Caco-2_BBe_) at 37°C or left uninfected. The extracellular media were collected, centrifuged (16,000 × *g*, 10 min 4°C), and subjected to trichloroacetic acid-induced protein precipitation, which recovered the proteins from the media. The presence of CTHD in them was analyzed by SDS-PAGE followed by Western blotting, using anti-CTHD antibodies (Table S4). To detect the CTHD in cell lysates, cells were lysed in 60 µL (HeLa) or 300 µL (Caco-2) SDS-PAGE sample buffer, and CTHD was detected by Western blotting. Protein loading was evaluated by probing the cell lysates with anti-α-tubulin antibodies (Table S4).

### The β-hexosaminidase activity (release) assay

The assay was performed essentially as described (107). HeLa (250,000), MDCK (800,000), and Caco-2_BBe_ (500,000) cells were seeded on a 6-well plate for 2 days, until reaching ~70% (HeLa), or full cell confluence (MDCK or Caco-2_BBe_). Cells were infected with pre-activated EPEC for 60 min (HeLa) or 120 min (MDCK) at 37°C. EPEC was pre-activated in high glucose DMEM lacking phenol red (Biological Industries 01-053-1A). Extracellular media (~1.2 mL) were collected and centrifuged (1,000 × *g*, 3 min, 4°C), and the supernatant was placed on ice. The cells were washed three times with ice-cold PBS, lysed in 1% NP-40 (in PBS), centrifuged (11,000 × *g*, 5 min 4°C), and the detergent soluble fraction, i.e., cell lysate, was stored on ice. The β-hexosaminidase activity assay was applied to 100 µL of the medium. For determination of the cellular content of β-hexosaminidase, cell lysates were diluted 1:10 with PBS, and 100 µL of the diluted lysates was taken to determine the enzyme activity. The samples were incubated for 15 min at 37°C with 15 µL of 6 mM 4-methylumbelliferyl-*N*-acetyl-β-d-glucosaminide substrate (Sigma, M2133) in sodium citrate-phosphate buffer, pH 4.5. The reaction was stopped by adding 30 µL of 2 M Na_2_CO_3_ and 1.1 M glycine, and the fluorescence was measured with a plate reader (Synergy H1 - Biotek - 1) at excitation 365 nm/emission 450 nm, gain 50. Results were expressed as enzyme activity measured in the cell medium (i.e., secreted enzyme) normalized to the enzyme activity measured in the cell medium and lysate (i.e., total enzyme activity).

### Analysis of PI uptake and Lamp-1-surface labeling of infected cells

#### Cell infection and imaging

Hela cells (80,000 cells) were seeded on coverslips (12 mm ɸ #1), placed on a 24-well dish, and allowed to adhere for 48 h. Caco-2_BBe_ cells (180,000 cells) were cultured on collagenated coverslips placed on a 24-well plate for 72 h until reaching full confluency. Cells were then infected with pre-activated bacteria or exposed to plain DMEM, supplemented with LB-lacking bacteria (uninfected) for 60 min at 37°C, as described (17, 19). Cells were then transferred to a metal plate on ice and treated with PI (50 µg/mL; Sigma P4170) for 5 min at 4°C, immediately after that washed with ice-cold PBS and incubated with mouse monoclonal anti-Lamp-1 primary antibodies (H4A3, DSHB, 10 µg/mL; Table S4) for 30 min at 4°C. Subsequently, cells were washed with ice-cold PBS, fixed with 4% paraformaldehyde for 60 min at 4°C, and stained with goat anti-mouse AlexaFluor488-labeled antibodies (Table S4) for 30 min at 22°C, as previously described (108). Then, the cells were post-fixed with 4% paraformaldehyde, permeabilized with saponin, and stained with Phalloidin-CF647 (Biotium, Cat. #00041) and DAPI, as described (17, 19). In experiments whose results are presented in Fig. 5A, cells were imaged using a Nikon Eclipse Ti microscope with an S Plan Fluor ELWD 20×/0.45 OFN22 Ph1 ADM MRH48230 objective, equipped with a Zyla 5.5 CMOS camera using the Nikon NIS Elements imaging software. The filters used in these experiments are listed in Table S5. For the experiments whose results are presented in Fig. 5B, the optical setup included an inverted NIKON Ti-E microscope equipped with an EMCCD iXon3 888 camera (Andor). Fluorescence excitation was provided with a Spectra X light engine (Lumencor). Table S6 contains the excitation bands and emission filters that were used for each fluorophore. A quad-band 405/488/561/640 nm beam-splitter (Chroma Technology Corp, VT, USA) was used together with the emission filters. Experiments were repeated independently twice, and two random fields were captured from each independent experiment.

### Determining the PI and Lamp-1 intensity level in single cells by microscopy

To optimize storage space and processing time, the acquired images were scaled to eight-bit depth, where pixel values ranged from 0 to 255. A top hat filter with a radius of 200 pixels was applied to remove the broad background from all images. Then, using the Cellpose software package (120), cell bodies were segmented based on phalloidin staining, and cell nuclei were identified based on DAPI staining, yielding cell body masks (Fig. S6). The mean Lamp-1 intensity was measured for each cell. Nuclei were segmented from the DAPI images using an OTSU threshold (121, 122) to create a region of interest set of the nuclei. Then, the mean PI intensity within the nuclei was measured. Segmented cell bodies containing no nuclei or more than one nucleus were disqualified. Cells and nuclei were filtered for outliers by fitting a linear regression line between the nuclei and the segmented-cell area, retaining only objects with residuals between the 5th and 95th percentiles of the overall population.

### Statistics

Results are presented as mean ± standard error (SE) of the mean. Unless otherwise indicated, the GraphPad Prism v. 8.4.3 software was used for statistical analysis and graphing. The two-tailed Student’s *t*-test was performed when only two groups were compared. A one-way ANOVA with Bonferroni’s test was applied to determine the statistical significance for multiple comparisons. The significance is indicated by asterisks (****P < 0.0005; ***P > 0.0005; **P < 0.005; *P > 0.005; ns, non-significant *P* > 0.05). A *P*-value < 0.05 indicates a statistically significant difference.

## SUPPLEMENTAL MATERIAL

Supplemental Figures mbio.01979-23-s0001.pdf Figures S1 to S6.

Legends mbio.01979-23-s0002.docx Supplemental figure legends and table titles.

Table S1 mbio.01979-23-s0003.pdf Description of EPEC strains used in the study.

Table S2 mbio.01979-23-s0004.pdf Plasmids.

Table S3 mbio.01979-23-s0005.pdf Primers.

Table S4 mbio.01979-23-s0006.pdf Antibodies.

Table S5 mbio.01979-23-s0007.pdf Nikon TI filters.

Table S6 mbio.01979-23-s0008.pdf Nikon TI-E filters.

ASM does not own the copyrights to Supplemental Material that may be linked to, or accessed through, an article. The authors have granted ASM a non-exclusive, world-wide license to publish the Supplemental Material files. Please contact the corresponding author directly for reuse.
