# Supplementary figures and images for: Enteropathogenic E. coli infection co-elicits lysosomal exocytosis and lytic host cell death
Source: mBio. 2023 Dec 1;14(6):e01979-23. doi: 10.1128/mbio.01979-23 (PMC10746156; doi:10.1128/mbio.01979-23)

Fig. S1

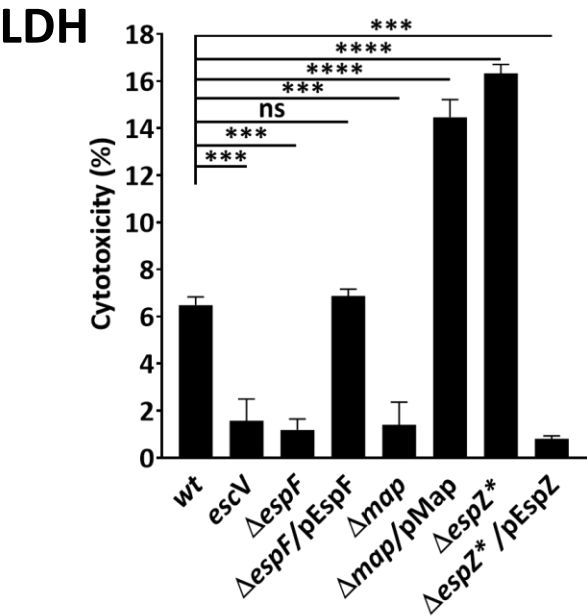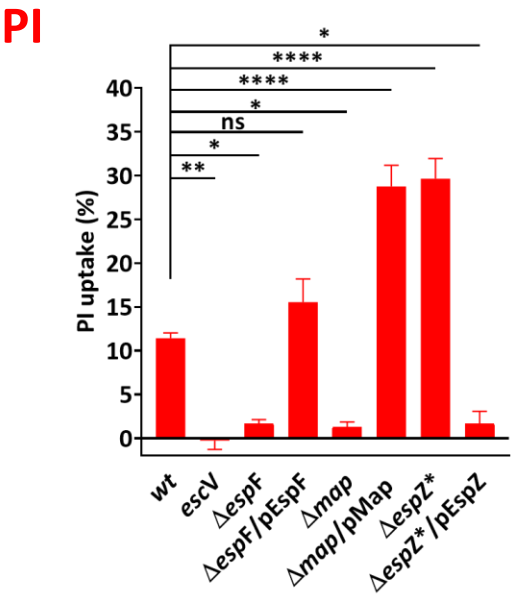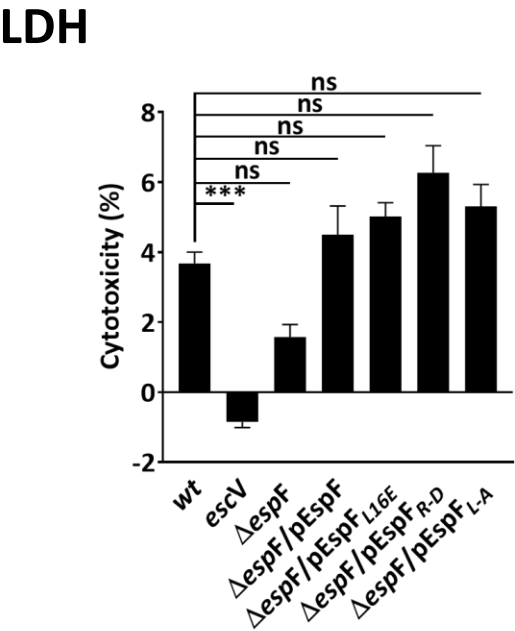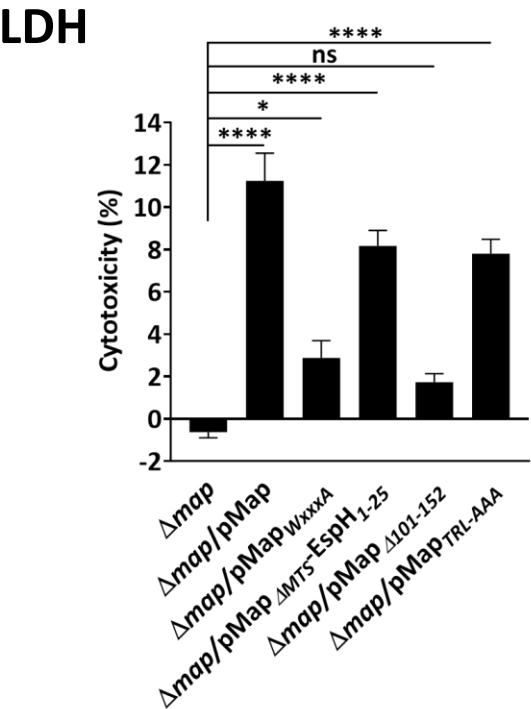

Fig. S2

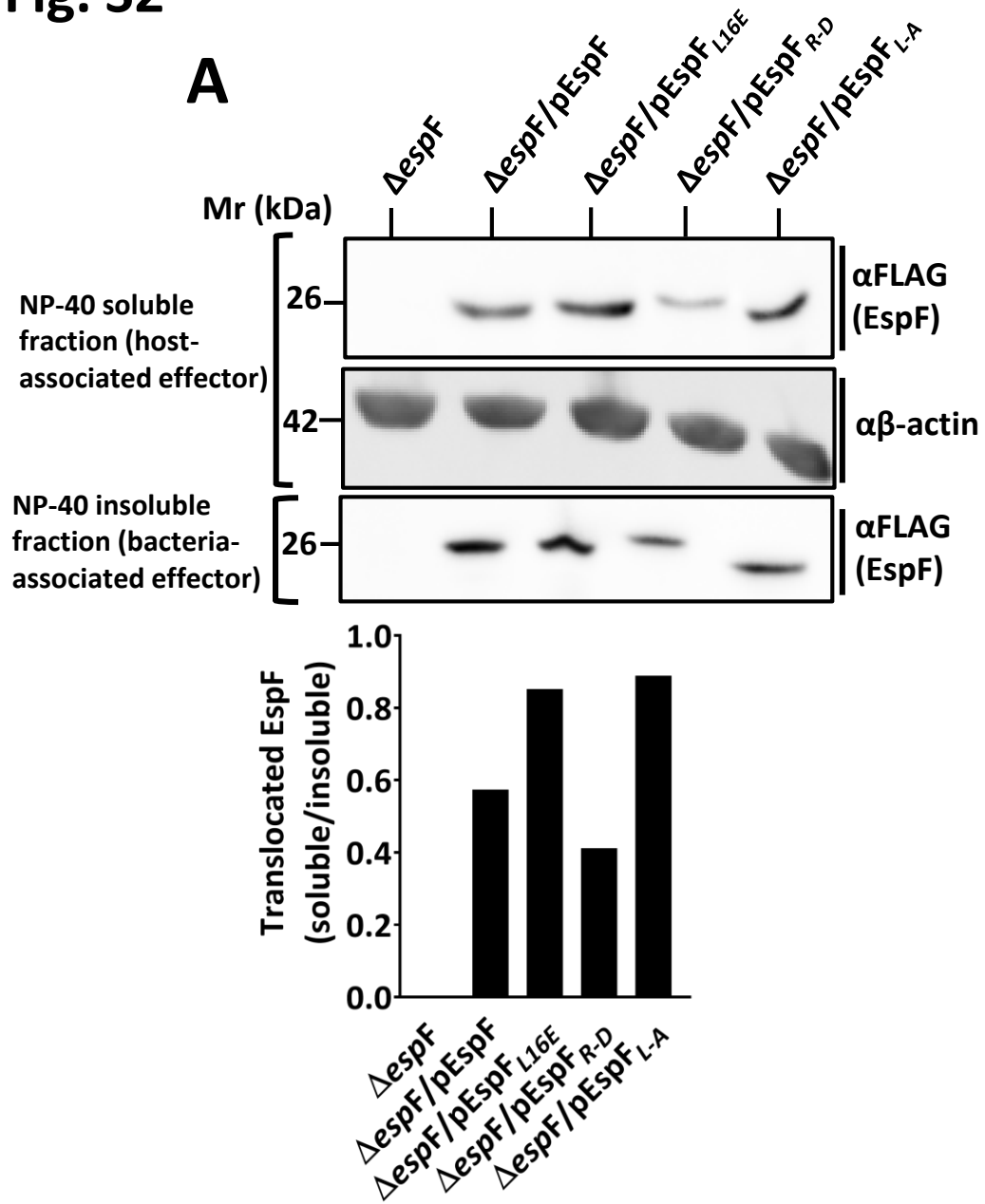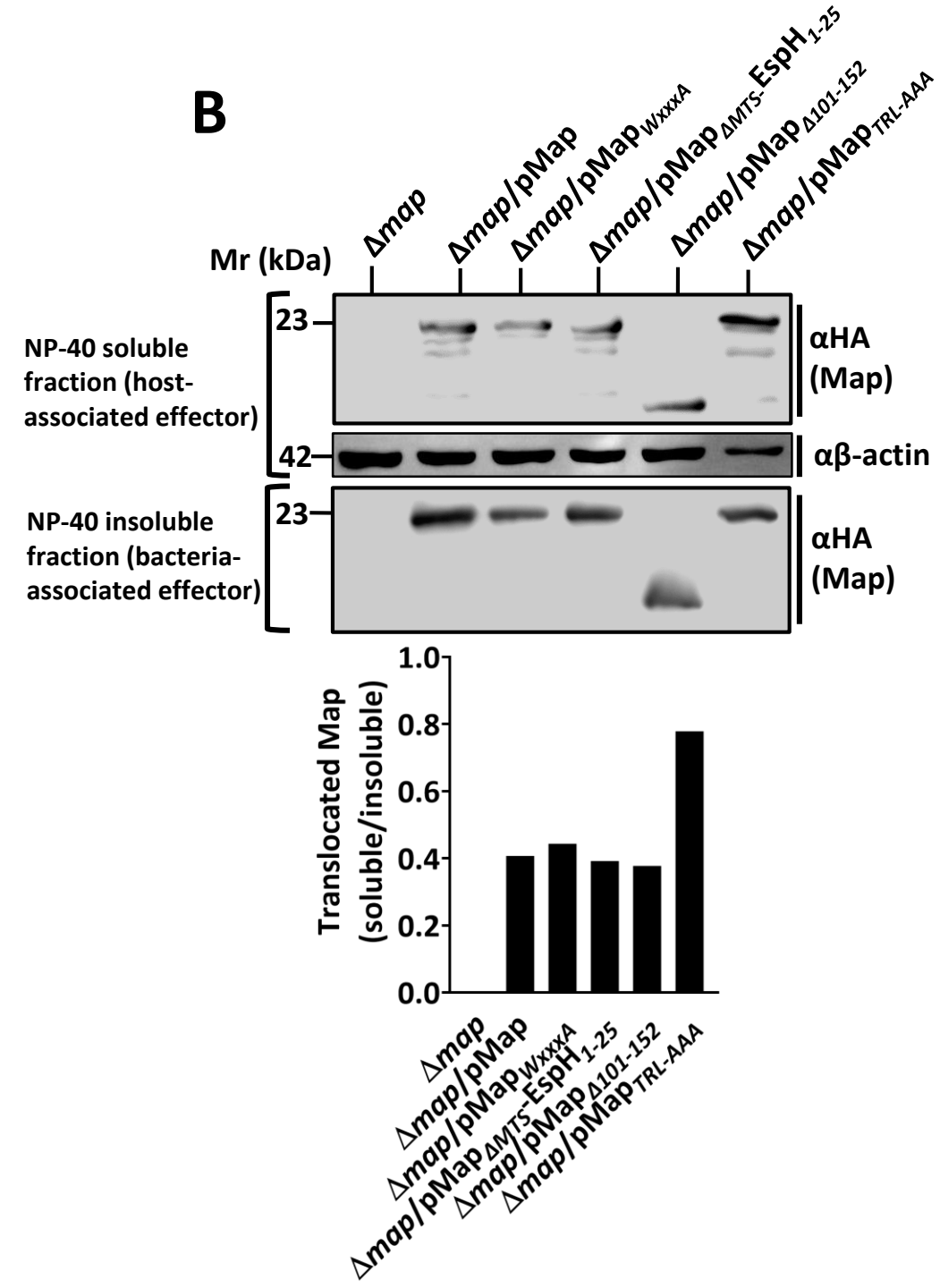

Fig. S3

**A**

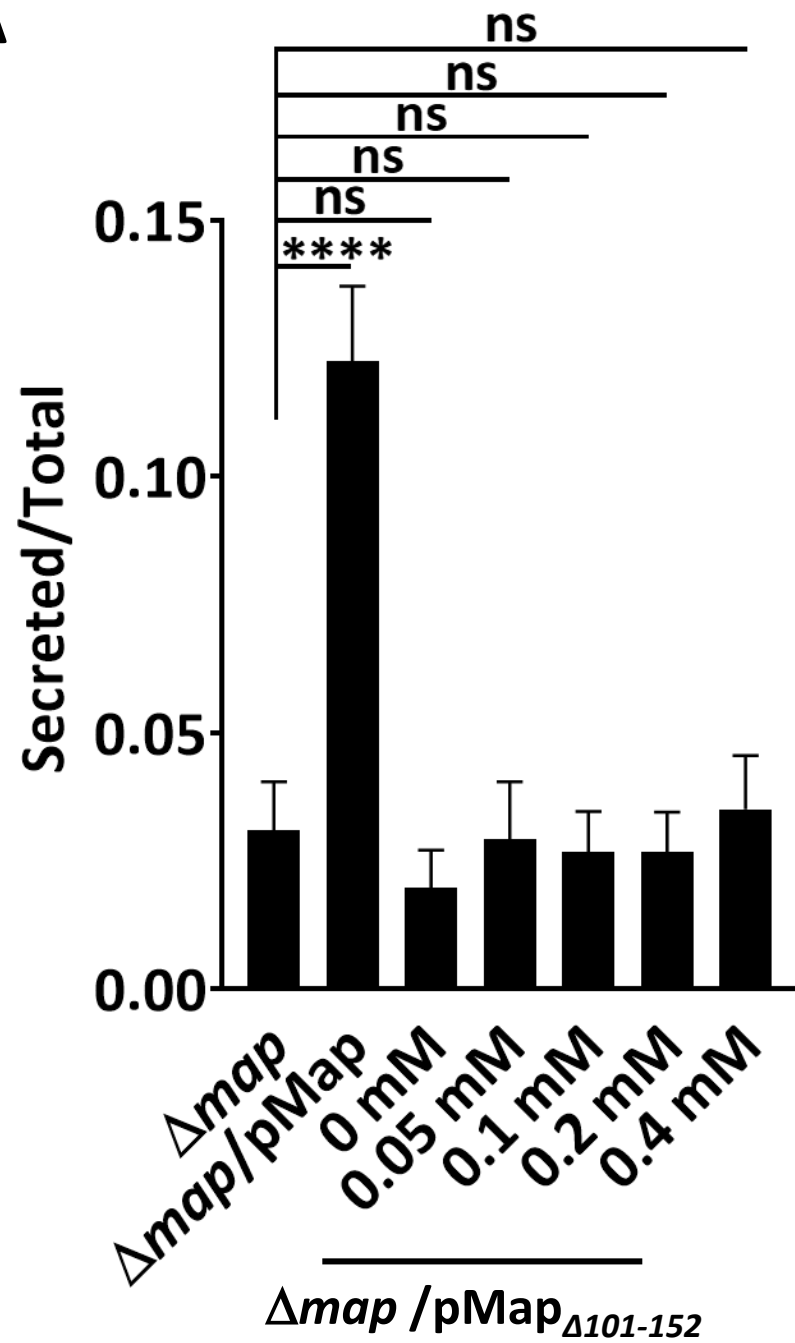

**B**

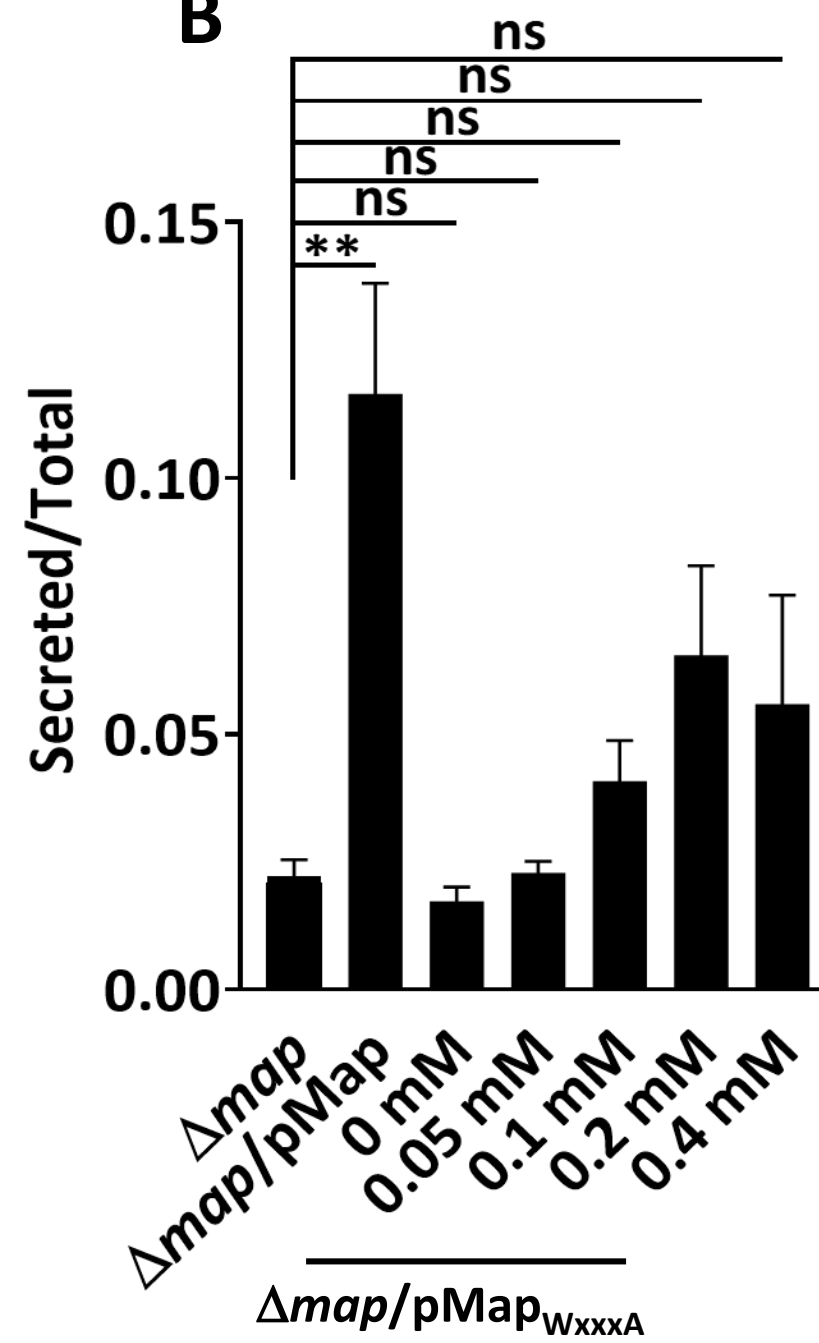

Fig. S4

A

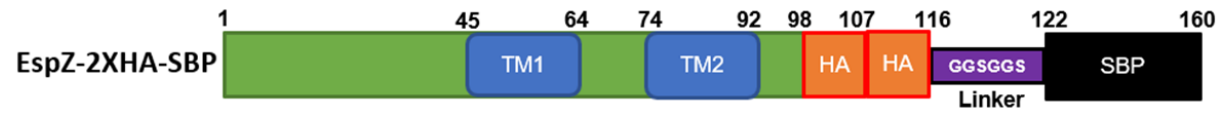

C

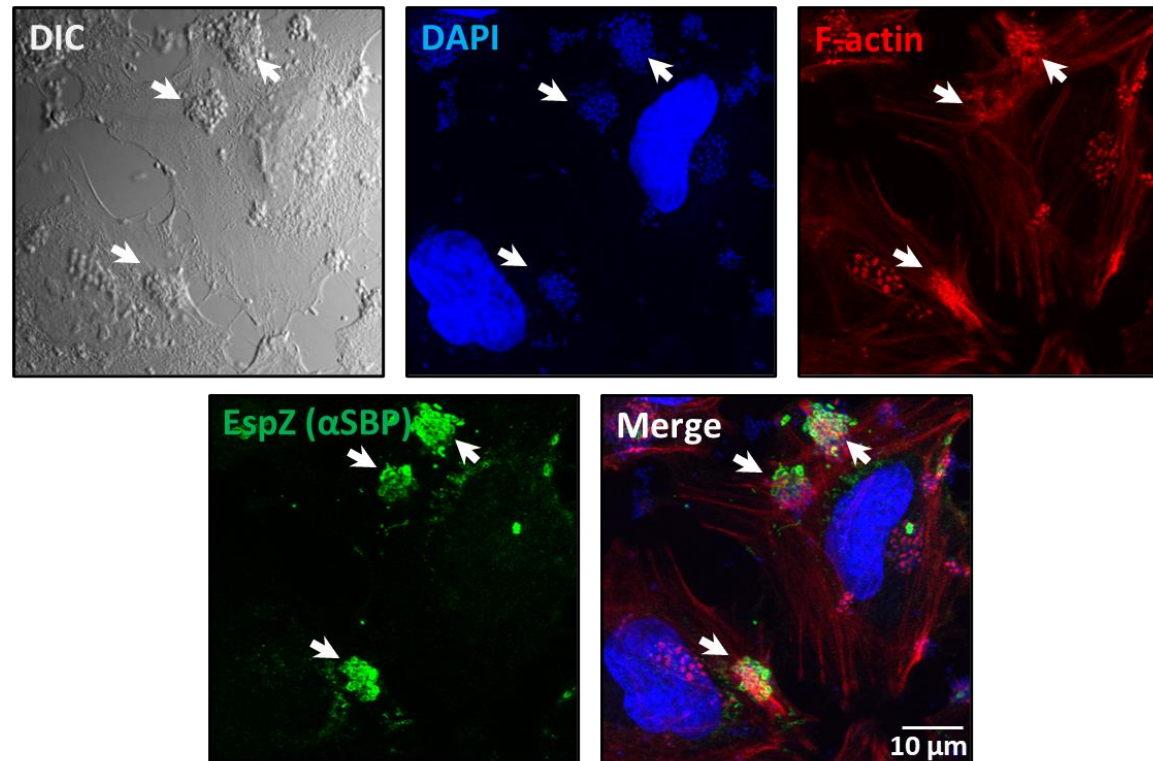

B

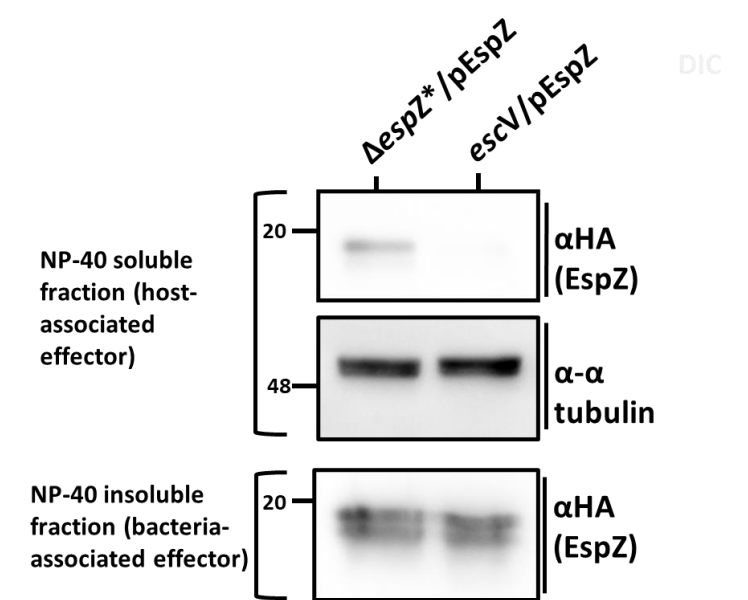

D

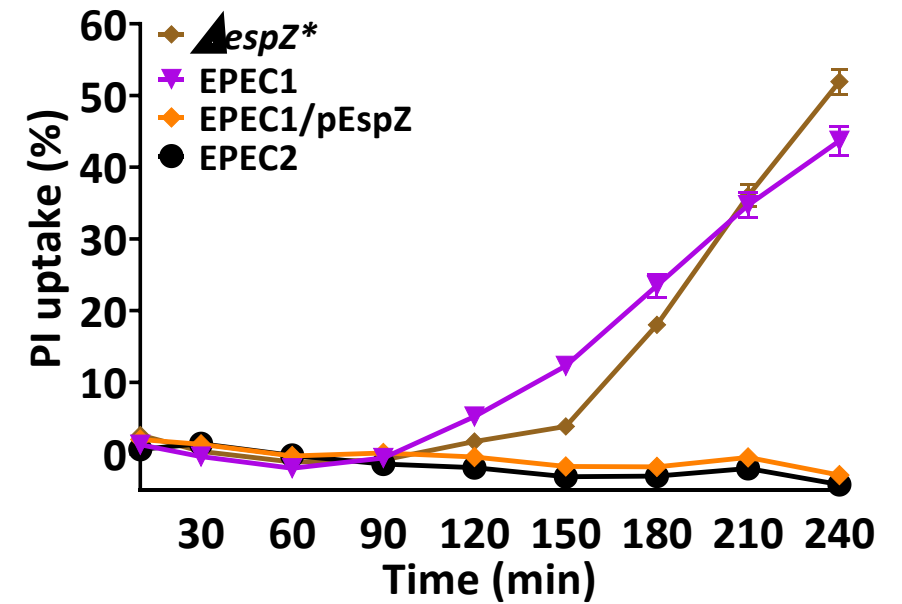

Fig. S5

## EPEC1

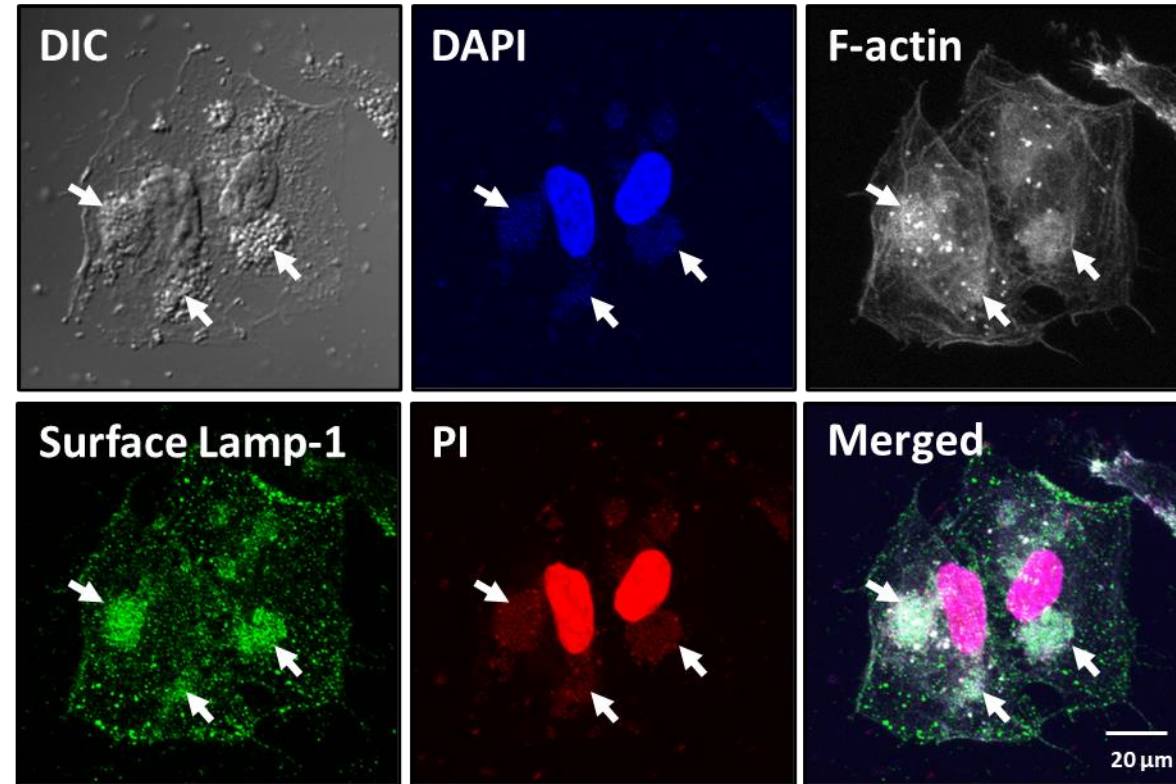

Fig. S6

HeLa

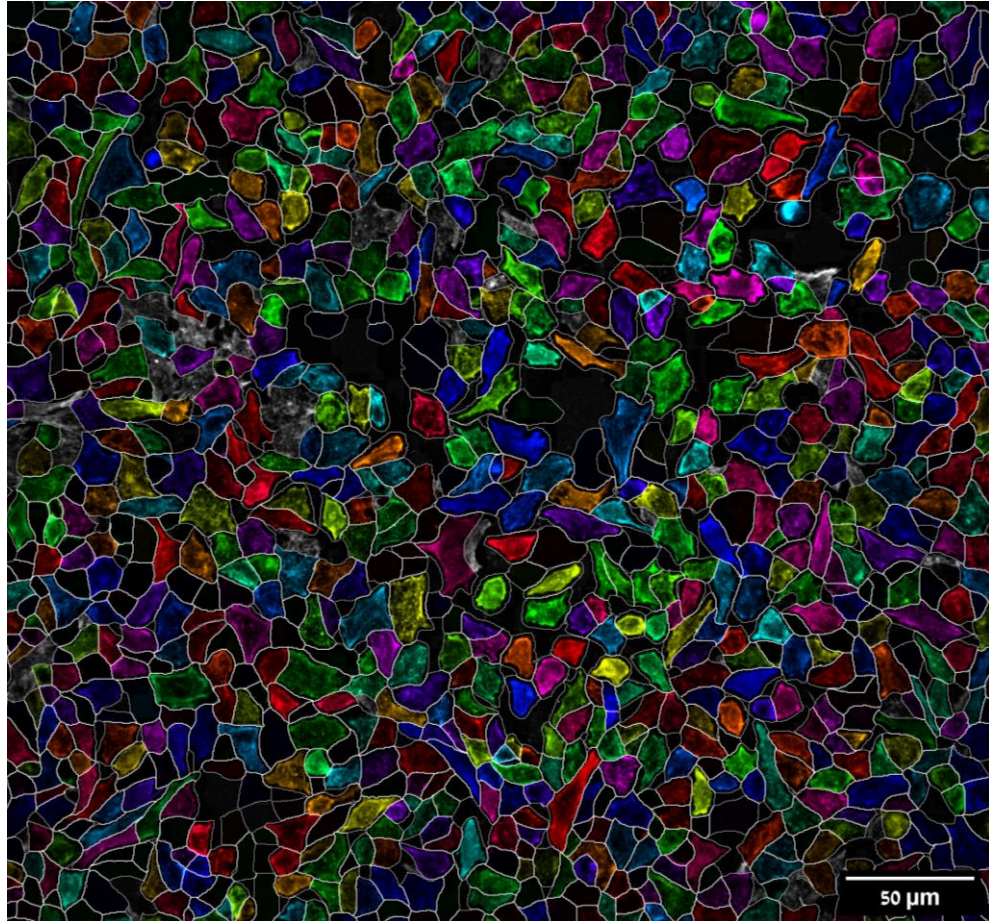

Caco-2<sub>BBe</sub>

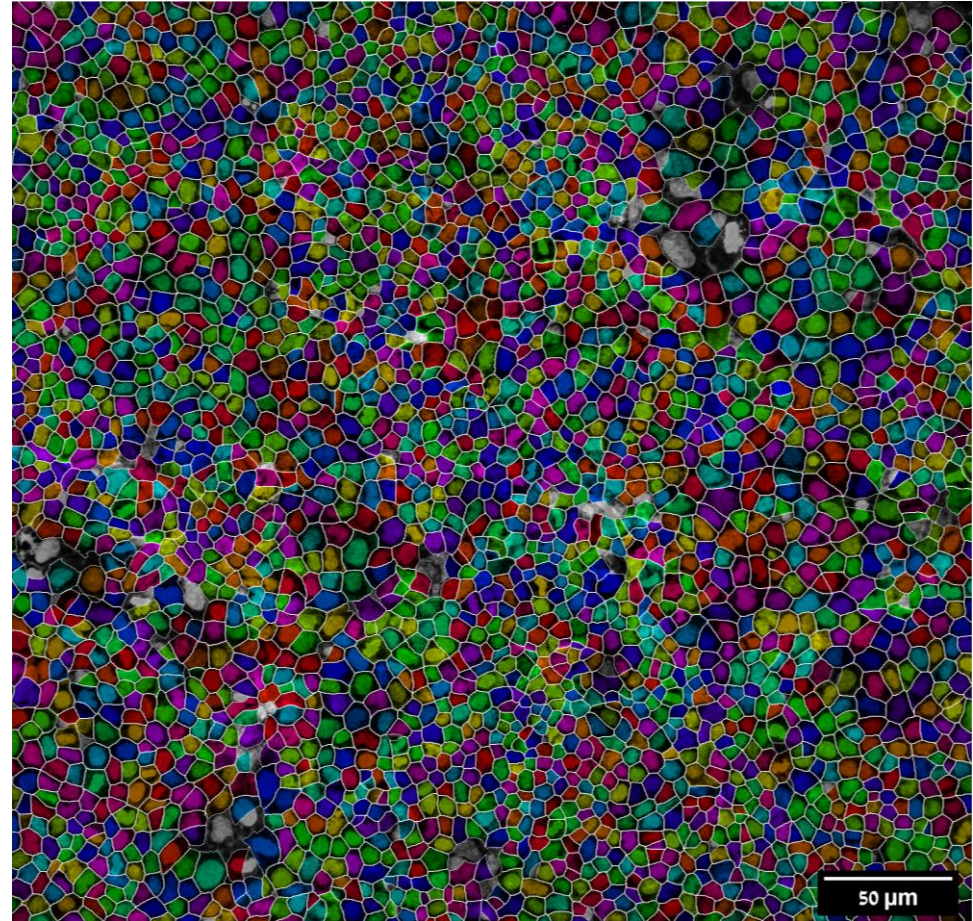

Supplement: Supplemental Figures — Figures S1 to S6. [file mbio.01979-23-s0001.pdf]
