# Supplementary material for: Enteropathogenic E. coli infection co-elicits lysosomal exocytosis and lytic host cell death
Source: mBio. 2023 Dec 1;14(6):e01979-23. doi: 10.1128/mbio.01979-23 (PMC10746156; doi:10.1128/mbio.01979-23)
Supplement: Legends — Supplemental figure legends and table titles. [file mbio.01979-23-s0002.docx]

**Supplementary Figure Legends**

**Fig. S1: Effects of Map, EspF, and EspZ on host cell cytotoxicity (LDH release) and PI entry.** HeLa cells were infected with the indicated EPEC strains. The LDH cytotoxicity and PI entry assays were performed, as described in Materials and Methods. Results are the mean ± SE from three independent experiments.

**Fig. S2: EspF and Map translocation into host cells.** HeLa cells were infected with the indicated *espF* (**A**), and *map* (**B**) EPEC strains and levels of effector translocation into the cells were performed, as previously described (1, 2). Following cell infection, cells were treated with an NP-40-containing buffer, and the detergent soluble and insoluble fractions were isolated by centrifugation. These fractions were then analyzed by SDS-PAGE followed by Western blotting and probed with anti-FLAG or anti-HA antibodies for detecting the EspF and Map effectors, respectively. While the NP-40 soluble fractions are considered to contain the translocated effectors, the NP-40 insoluble fractions comprise the bacterial-associated effectors. Probing with an anti-β-actin antibody is used to evaluate the cell lysate protein loading level. Representative gels (**upper**) and protein band quantifications (**lower**) are presented.

**Fig. S3**: **β-hexosaminidase secretion upon EPEC-**Δ***map*+Map*_Δ101-152_*** or **EPEC-**Δ***map*+Map*_WxxxA_* infection under increasing IPTG concentrations.** HeLa cells were infected with the indicated EPEC strains. The expression of Map in EPEC-Δ*map*/pMap was induced with 0.2 mM IPTG. The term Map*_Δ101-152_* and Map*_WxxxA_* was induced with IPTG concentrations ranging from 0 to 0.4 mM. β-hexosaminidase secretion was measured, as described in Materials and Methods. Results are the mean ± SE from 3 independent experiments.

**Fig. S4:** **Characterizing EspZ translocation and protection against cell death conferred by EPEC-Δ*espZ*. (A) *Illustration of the EspZ-2xHA-SBP construct*.** The amino acids are numbered in sequence, starting with the N-terminus of the protein. The two predicted transmembrane (TM) domains, HA tags, GGSGGS linker, and SBP are indicated. **(B)** ***EspZ translocation***. HeLa cells were infected for 60 min with the indicated EPEC strains, and the effector translocation assay was performed as described in **Fig. S4B**. Still, anti-HA and anti-tubulin antibodies were used to detect the effector and protein loading levels, respectively. A representative image from two independent experiments is shown. **(C)** ***Imaging of translocated EspZ*.** HeLa cells were infected for 45 min with EPEC-Δ*espZ**+EspZ, fixed, permeabilized, and stained with DAPI (Sigma #D9542) and TR-phalloidin (Life Technologies, #T7471) for visualizing bacterial and host cell DNA, and F-actin-rich pedestals, respectively. For imaging EspZ, cells were immunostained with anti-HA antibodies followed by fluorescent (Alexa488) secondary antibodies. Cell processing and imaging were done by confocal microscopy, as described in Materials and Methods. Representative differential interference contrast (DIC), DAPI, F-actin, and EspZ images are shown. Arrows point toward infecting EPEC microcolonies. **(D)** ***Effects of EspZ on lytic cell death***. HeLa cells were infected with the indicated EPEC strains and lytic cell death, using the PI-uptake (left) and LDH release cytotoxicity assays (right) were applied, as described in Materials and Methods. Results are the mean ± SE from 3 to 6 independent experiments.

**Fig. S5: Surface Lamp-1 clustered at infection sites of EPEC1 infected cells.** HeLa cells were infected with pre-activated EPEC1 for 2 hrs, exposed to PI, and stained with anti-Lamp-1 antibodies using the surface labeling procedure described in Materials and Methods. Cells were then fixed, permeabilized, and stained with DAPI and TR-phalloidin to visualize DNA (bacterial microcolonies and host nuclei) and the host F-actin, respectively. Arrows indicate bacterial infection sites. Surface Lamp-1 clusters at infection sites are visualized.

**Fig. S6: Identification of individual HeLa and Caco-2_BBe_ cell bodies.** Expected outputs of the Cellpose software, used to segment HeLa and Caco-2_BBe_ cell bodies (see Materials and Methods), are shown. The predicted cell boundaries are indicated with white lines, and the cell body masks (i.e., fluorescently labeled cells) are pseudo-colored**.** Note that some cells are outlined but not colored. Therefore, these cells did not fluoresce and were excluded from our calculations (see Materials and Methods).

**Supplementary Tables**

**Table S1:** EPEC strains

**Table S2:** Plasmids

**Table S3:** Primers

**Table S4:** Primary and secondary antibodies

**Table S5:** Excitation and emission filters used for fluorophores imaged on the Nikon Ti microscope. Filters specified as center-wavelength/bandwidth

**Table S6:** Excitation bands and emission filters used for fluorophores imaged on the Nikon Ti-E microscope. Filters specified as center-wavelength/bandwidth.

**References**

1. Ramachandran RP, Spiegel C, Keren Y, Danieli T, Melamed-Book N, Pal RR, Zlotkin-Rivkin E, Rosenshine I, Aroeti B. 2020. Mitochondrial Targeting of the Enteropathogenic Escherichia coli Map Triggers Calcium Mobilization, ADAM10-MAP Kinase Signaling, and Host Cell Apoptosis. mBio 11.

2. Kassa EG, Zlotkin-Rivkin E, Friedman G, Ramachandran RP, Melamed-Book N, Weiss AM, Belenky M, Reichmann D, Breuer W, Pal RR, Rosenshine I, Lapierre LA, Goldenring JR, Aroeti B. 2019. Enteropathogenic Escherichia coli remodels host endosomes to promote endocytic turnover and breakdown of surface polarity. PLOS Pathogens 15:e1007851.
