# Supplementary material for: Enteropathogenic E. coli infection co-elicits lysosomal exocytosis and lytic host cell death
Source: mBio. 2023 Dec 1;14(6):e01979-23. doi: 10.1128/mbio.01979-23 (PMC10746156; doi:10.1128/mbio.01979-23)
Supplement: Table S1 — Description of EPEC strains used in the study. [file mbio.01979-23-s0003.pdf]

Table S1: EPEC strains (all mutant strains are derivatives of E2348/69)

Strain name & number	Description	Source/References	Comments
E2348/69 (*wt*)<br>BA250	EPEC-*wt::Strp*^{r}<br>EPEC-*wt* isolate, serotype O127:H6	I. Rosenshine (HUJI); originally from J. Kaper (UMD) (1)	EPEC-*wt* isolate
SN191(*escV*)<br>BA253	*escV*::*miniTn5Kn*^{r}	I. Rosenshine (HUJI) (2)	EPEC mutated in the T3SS
UMD874 (Δ*esp*F)<br>BA 1063	Δ*esp*F*::Kn*^{r}	M.S. Donnenberg (VCU) (3, 4)	LEE effector Δ*espF* mutant
ICC202 (Δ*map*)<br>BA 1079	Δ*map::Kn*^{r}	G. Frankel (Imperial College) (5)	LEE effector Δ*map* mutant
BA1198	Δ*map,espF:: Kn*^{r}*+Cm*^{r}	I. Rosenshine (HUJI) (6)	*map,espF* double mutant
BA1211	Δ*map,espF/*pEspF:: *Knr+Cm*^{r}*+Amp*^{r}	(6)	*map,espF* double mutant strain complemented with a C-terminally Flag-tagged EspF$_{wt}$ encoding plasmid

BA1212	Δ*map,*espF/pMap:: *Knr+Cm*^{r}*+Amp*^{r}	(6)	*map, espF* double mutant strain complemented with a psA10-Map$_{wt}$ encoding plasmid
XT111 (Δ*espH*) BA 1101	Δ*espH::Kn*^{r}	I. Rosenshine (HUJI) (7)	LEE effector Δ*espH* mutant
SE1207 (Δ*espG1*, Δ*espG2*) BA 132	Δ*espG1,espG2::Kn*^{r}	M.S. Donnenberg (VCU); originally from J. Kaper (UMD) (8)	LEE effector Δ*espG1* and non-LEE effector Δ*espG2* mutant
EM3458 (Δ*tir*) BA 251	Δ*tir::Cm*^{r}	I. Rosenshine (HUJI) (9)	LEE effector Δ*tir* mutant
BA 1143	Δ*espZ**::*Kn*^{r}	I. Rosenshine (HUJI)	EPEC deleted in IE6, PP4 and *espZ*
BA 1254	Δ*espF*+EspF; *Kn*^{r}*+Amp*^{r}	(10)	EPEC-Δ*espF* strain complemented with a pSA10-EspF$_{wt}$ plasmid
BA1255	Δ*esp*F+EspF$_{L16E}$::*Kn*^{r}*+Amp*^{r}	This study	EPEC-Δ*esp*F complemented with

			a pSA10-EspF$_{L16E}$ encoding plasmid. The L16E mutation abolishes mitochondrial targeting of EspF.
BA1266	Δ*esp*F+EspF$_{R-D}$::Kn^r+Amp^r	This study	EPEC-Δ*esp*F complemented with a psA10-EspF$_{R-D}$ encoding plasmid. The Arginine (R) to Aspartic acid mutation of each of the three PRR domains of EspF abolishes its binding to host SNX9.
BA 1267	Δ*esp*F+EspF$_{L-A}$::Kn^r+Amp^r	This study	EPEC-Δ*esp*F complemented with a pSA10-EspF$_{R-D}$ encoding plasmid. The Leucine (L) to Alanine (A) mutation

			of each of the three PRR domains of EspF abolishes its binding to ho N-WASP.
BA1178	Δ*map*+Map$_{wt}$:: *Amp*^{r}	(6)	EPEC-Δ*map* strain complemented with a pSA10-Map$_{wt}$ plasmid
BA 1202	Δ*map*+Map$_{wxxxA}$:: *Kn*^{r}+*Amp*^{r}	(6)	EPEC-Δ*map* complemented with a pSA10-MapwxxxA encoding plasmid. This mutation abolishes the RhoGEF activity of Map.
BA1222	Δ*map*+Map$_{\Delta MTS\text{-}EspH_{1\text{-}25}}$ :: *Amp*^{r}	(6)	EPEC-Δ*map* complemented with a pSA10-Map$_{\Delta MTS}$EspH$_{1\text{-}25}$ encoding plasmid. This mutation abolishes

			mitochondrial targeting of Map.
BA 1223	Δ*map*+Map$_{\Delta 101\text{-}152}$::*Amp*^{r}	(6)	EPEC-Δ*map* complemented with a pSA10-Map$_{\Delta 101\text{-}152}$ encoding plasmid. This mutation, which detects the MTR of Map, disables it to alter mitochondrial morphology and induces cytotoxicity.
BA 1257	Δ*map*+Map$_{TRL\text{-}AAA}$:: *Kn*^{r}+*Amp*^{r}	This study	EPEC-Δ*map* complemented with a pSA10-Map$_{TRL\text{-}AAA}$ encoding plasmid. This Map mutant is deficient in binding PDZ type I proteins of the host (11).
BA 1224	Δ*esp*Z*+EspZ-2xHA-SBP:: *Kn*^{r}+*Amp*^{r}	This study	EPEC-Δ*esp*Z* complemented with

			pSA10-EspZ-2xHA-SBP encoding plasmid
BA 1251	*escV*-EspZ-2xHA-SBP::*Kn*^{r}+*Amp*^{r}	This study	EPEC-*escV* complemented with a pSA10-EspZ-2xHA-SBP encoding plasmid
BA 1269	EPEC0::*Strep*^{r}	(12)	EPEC0 is effector less
BA 1270	EPEC1::*Strep*^{r}	(12)	EPEC1 expresses only Tir
BA 1273	EPEC1+EspZ-2xHA-SBP::*Strep*^{r}+*Amp*^{r}	This study	EPEC1 complemented with a pSA10-EspZ-2xHA-SBP encoding plasmid
BA 1271	EPEC2::*Strep*^{r}	(12)	EPEC2 expresses only Tir and EspZ

References

1. Levine, M. M., Bergquist, E. J., Nalin, D. R., Waterman, D. H., Hornick, R. B., Young, C. R., and Sotman, S. (1978) Escherichia coli strains that cause diarrhoea but do not produce heat-labile or heat-stable enterotoxins and are non-invasive. *Lancet* **1**, 1119-1122

2. Nadler, C., Shifrin, Y., Nov, S., Kobi, S., and Rosenshine, I. (2006) Characterization of enteropathogenic Escherichia coli mutants that fail to disrupt host cell spreading and attachment to substratum. *Infect Immun* **74**, 839-849
3. Crane, J. K., McNamara, B. P., and Donnenberg, M. S. (2001) Role of EspF in host cell death induced by enteropathogenic Escherichia coli. *Cell Microbiol* **3**, 197-211
4. McNamara, B. P., Koutsouris, A., O'Connell, C. B., Nougayrede, J. P., Donnenberg, M. S., and Hecht, G. (2001) Translocated EspF protein from enteropathogenic Escherichia coli disrupts host intestinal barrier function. *J Clin Invest* **107**, 621-629
5. Wong, A. R., Clements, A., Raymond, B., Crepin, V. F., and Frankel, G. (2012) The interplay between the Escherichia coli Rho guanine nucleotide exchange factor effectors and the mammalian RhoGEF inhibitor EspH. *MBio* **3**
6. Ramachandran, R. P., Spiegel, C., Keren, Y., Danieli, T., Melamed-Book, N., Pal, R. R., Zlotkin-Rivkin, E., Rosenshine, I., and Aroeti, B. (2020) Mitochondrial Targeting of the Enteropathogenic Escherichia coli Map Triggers Calcium Mobilization, ADAM10-MAP Kinase Signaling, and Host Cell Apoptosis. *MBio* **11**
7. Tu, X., Nisan, I., Yona, C., Hanski, E., and Rosenshine, I. (2003) EspH, a new cytoskeleton-modulating effector of enterohaemorrhagic and enteropathogenic Escherichia coli. *Mol Microbiol* **47**, 595-606
8. Elliott, S. J., Krejany, E. O., Mellies, J. L., Robins-Browne, R. M., Sasakawa, C., and Kaper, J. B. (2001) EspG, a novel type III system-secreted protein from enteropathogenic Escherichia coli with similarities to VirA of Shigella flexneri. *Infection and immunity* **69**, 4027-4033
9. Mills, E., Baruch, K., Aviv, G., Nitzan, M., and Rosenshine, I. (2013) Dynamics of the type III secretion system activity of enteropathogenic Escherichia coli. *MBio* **4**
10. Kassa, E. G., Zlotkin-Rivkin, E., Friedman, G., Ramachandran, R. P., Melamed-Book, N., Weiss, A. M., Belenky, M., Reichmann, D., Breuer, W., Pal, R. R., Rosenshine, I., Lapierre, L. A., Goldenring, J. R., and Aroeti, B. (2019) Enteropathogenic Escherichia coli remodels host endosomes to promote endocytic turnover and breakdown of surface polarity. *PLOS Pathogens* **15**, e1007851
11. Simpson, N., Shaw, R., Crepin, V. F., Mundy, R., FitzGerald, A. J., Cummings, N., Straatman-Iwanowska, A., Connerton, I., Knutton, S., and Frankel, G. (2006) The enteropathogenic Escherichia coli type III secretion system effector Map binds EBP50/NHERF1: implication for cell signalling and diarrhoea. *Mol Microbiol* **60**, 349-363
12. Cepeda-Molero, M., Berger, C. N., Walsham, A. D. S., Ellis, S. J., Wemyss-Holden, S., Schuller, S., Frankel, G., and Fernandez, L. A. (2017) Attaching and effacing (A/E) lesion formation by enteropathogenic E. coli on human intestinal mucosa is dependent on non-LEE effectors. *PLoS Pathog* **13**, e1006706
