# Supplementary material for: Enteropathogenic E. coli infection co-elicits lysosomal exocytosis and lytic host cell death
Source: mBio. 2023 Dec 1;14(6):e01979-23. doi: 10.1128/mbio.01979-23 (PMC10746156; doi:10.1128/mbio.01979-23)
Supplement: Table S2 — Plasmids. [file mbio.01979-23-s0004.pdf]

**Table S2: Plasmids**

| Plasmid name | Description | Source/Reference |
|---|---|---|
| pAA6284 | pSA10 derivative encoding EspH-6xHis-SBP; Amp$^r$ | (1) |
| pJN61-EspF-FLAG | A pTrc99A-based vector encoding EspF (EPEC O127:H6 E2348/69) tagged with C-terminal FLAG (EspF-FLAG) | (2) |
| pSA10-EspF-FLAG (BA866) | pSA10 derivative encoding EspF-FLAG (EPEC 0127:H6 E2348/69) | This study |
| pSA10-EspF$_{L16E}$ (BA864) | pSA10 derivative encoding EspF-FLAG in which Leucine (L) at position 16 has been substituted for Glutamic acid (E). This mutation inhibits EspF targeting to mitochondria. | This study |
| pSA10-EspF$_{R\text{-}D}$ (BA867) | pSA10 derivative encoding EspF-FLAG in which Arginines (R) in each of the three SNX-9 binding motifs of EspF has been substituted for Aspartic acid (D). This mutation inhibits host SNX-9 binding to EspF. | This study |
| pSA10-EspF$_{L\text{-}A}$ (BA903) | pSA10 derivative encoding EspF-FLAG in which Leucines (L) in each of the three N-WASP binding motifs of EspF has been substituted for Alanine (A). This mutation inhibits EspF binding to N-WASP. | This study |
| pSA10- Map (BA564) | pSA10 derivative encoding HA-tagged Map (EPEC 0127:H6 E2348/69) (Map-HA). | (3, 4) |
| pSA10 - MapTRL-AAA (BA862) | pSA10 derivative encoding Map-HA in which the C-terminal Threonine Arginine Leucine (TRL 201-203) PDZ type I binding residues have been mutated to Alanines (AAA). This mutation abrogates Map binding to host | This study |

| | | |
|---|---|---|
| | proteins containing PDZ type I domains. (5) (5) | |
| pSA10-EspZ-2xHA-SBP (BA840) | pSA10 derivative encoding EspZ C-terminally tagged sequentially with two HA tags and streptavidin binding peptide (SBP) | (6) |
| mCherry-EspZ BA591 | EspZ C-terminally fused to mCherry | From Prof. Ilan Rosensine (HUJI) |

**References**


1. Ramachandran RP*, et al.* (2018) EspH Suppresses Erk by Spatial Segregation from CD81 Tetraspanin Microdomains. *Infection and immunity* 86(10):00303-00318.
2. McNamara BP*, et al.* (2001) Translocated EspF protein from enteropathogenic Escherichia coli disrupts host intestinal barrier function. *J Clin Invest* 107(5):621-629.
3. Simpson N*, et al.* (2006) The enteropathogenic Escherichia coli type III secretion system effector Map binds EBP50/NHERF1: implication for cell signalling and diarrhoea. *Mol Microbiol* 60(2):349-363.
4. Berger CN*, et al.* (2012) EspZ of enteropathogenic and enterohemorrhagic Escherichia coli regulates type III secretion system protein translocation. *MBio* 3(5).
5. Dean P & Kenny B (2009) The effector repertoire of enteropathogenic E. coli: ganging up on the host cell. *Curr Opin Microbiol* 12(1):101-109.
6. Haritan N*, et al.* (2023) Topology and function of translocated EspZ. *MBio*:e0075223.
