# Supplementary material for: Enteropathogenic E. coli infection co-elicits lysosomal exocytosis and lytic host cell death
Source: mBio. 2023 Dec 1;14(6):e01979-23. doi: 10.1128/mbio.01979-23 (PMC10746156; doi:10.1128/mbio.01979-23)
Supplement: Table S3 — Primers. [file mbio.01979-23-s0005.pdf]

Table S3. List of primers and their usage

S. No.	Name	Sequence	Usage
1F'	EspF FL F'	ATGCTTAATGGAATTAGTAACGCTGCTTC	Generation of pSA10-EspF$_{wt}$-FLAG
2R'	EspF plus FLAG R'	TCAATCCTTATCATCATCATCCTTATAATCCCCT TTCTTCGATTGCTCATAGGC	Generation of pSA10-EspF$_{wt}$-FLAG
3F'	pSA10 lin GA EspF F'	GATGATGATGATAAGGATTGAACAATTCGCG CGCGAAGGCG	Generation of pSA10-EspF$_{wt}$-FLAG
4R'	R' pSA10 lin tail EspF	GTTACTAATTCCATTAAGCATATTCTGTTTCCT GTGTGAAATTGTTATCCG	Generation of pSA10-EspF$_{wt}$-FLAG
5F'	F` lin L16E	GCG GCA GGA AGT AGG TAT CGC AAG TCG	Generation of pSA10-EspF$_{L16E}$-FLAG
6R'	Lin L16E EspF R`	CGC GCG AAT TGT TCA ATC CTT ATC ATC ATC ATC	Generation of pSA10-EspF$_{L16E}$-FLAG
7	R2D gBlock	CTT CAT TTA CTC CCT CTG ACC CAG CGC CGC CGC CAC CAA CCT CTGGAC AGG CAT CCG GCG CAA GCC GAC CTT TAC CAC CCA TTG CAC AAG CAT TAA AAG ATC ACT TAG CGG CCT ATG AAC TAT CTA AAG CGT CTG AAA CTG TAA ACT TTA AAC CAA CCG ACC CGG CAC CGC CAC CAC CAA CAA GTG GTC AAG CAT CCG GGG CAT CCC GAC CTT TGC CGC CCA TTG CAC AGG CTT TAA AGG ATC ATT TGG CTG CCT ATG AAT TAT CGA AAG CGT CTG AGA CTG TAA GCT TCA AGC CAA CCG ACC AGG CAC CAC CGC CAC CGA CAA GTG GCC AGG CAT CCG GTC CTG GTG GAC TAC CGC CCC TTG CAC AGG CAC TAA AAG ATC ATT TAG CTG CCT ATG AGC AAT CGA AGA AAG GGG ATT ATA AGG ATG ATG ATG ATA AGG AT	Generation of pSA10-EspF$_{R-D}$-FLAG
8F'	F R2D lin	GAT GAT GAT GAT AAG GAT TGA ACA ATT CGC	Generation of pSA10-EspF$_{R-D}$-FLAG

9R'	R R2D lin	AGA GGG AGT AAA TGA AGT CAC CTG GCT G	Generation of pSA10-EspF$_{R\text{-}D}$-FLAG
10	EspF L2A gblock new	GCA CAA GCG CTG AAA GAC CAC GCA GCT GCT TAT GAA CTT TCG AAG GCTTCA GAA ACA GTC AAT TTC AAG CCA ACG CGC CCT GCT CCT CCA CCACCT ACT TCA GGG CAG GCA TCG GGA GCA TCA CGC CCT CTG CCT CCA ATTGCA CAG GCC TTA AAG GAC CAT GCT GCT GCA TAT GAG CTT AGT AAG GCCTCG GAA ACC GTA AGT TTT AAA CCT ACC CGC CAG GCC CCT CCG CCACCT ACT AGC GGG CAG GCG TCG GGA CCT GGT GGA CTG CCC CCG TTG GCA CAG GCA CTT AAA GAT CAT GCT GCT GCC TAT GAG CAG AGT AAG AAG GGT GAC TAC AAA GAT GAC GAC GAT AAA GAC TGA	Generation of pSA10-EspF$_{L\text{-}A}$-FLAG
11F'	L2A F` 070322	ATG ACG ACG ATA AAG ACT GAA CAA TTC GCG CGC GAA GGC	Generation of pSA10-EspF$_{L\text{-}A}$-FLAG
12R'	L2A R` 070322	TGG TCT TTC AGC GCT TGT GCA ATG GGC GGT AAA GGT CGG G	Generation of pSA10-EspF$_{L\text{-}A}$-FLAG
13F'	F' lin TRL2AAA	GGAGCACAGGATGCGGCTGCGTAGGTGAACAATTCGCG	Generation of pSA10 -Map$_{TRL\text{-}AAA}$
14R'	Psa10 Map R'	ATTGCCGTTGGACTAAACATATTCTGTTTCCTGTG	Generation of pSA10 -Map$_{TRL\text{-}AAA}$
15F'	Map F' lin Psa10	ATGTTTAGTCCAACGGCAATGG	Generation of pSA10 -Map$_{TRL\text{-}AAA}$
16R'	R' lin QD Map	ATCCTGTGCTCCAGCGTAGTCTGGG	Generation of pSA10 -Map$_{TRL\text{-}AAA}$
17F'	pSA10-SBP F' linear GA	ATGGACGAAAAAACCACCGGTT	Generation of pSA10-EspZ-2xHA-SBP
18R'	pSA10-EspZ tail R' linear GA	CTTAAATTTGCTGCTTCCATAATTCTGTTTCCTGTGTGAAATTGTTATCCG	Generation of pSA10-EspZ-2xHA-SBP
19F'	EspZ F' GA	ATGGAAGCAGCAAATTTAAGCCCTTC	Generation of pSA10-EspZ-2xHA-SBP

20R'	EspZ R' GA	GGCATATTTCATCGCTAATCCGCCG	Generation of pSA10-EspZ-2xHA-SBP
21F'	2xHA EspZ tail F' GA	GATTAGCGATGAAATATGCCTATCCATACGATGTGCCTGATTATGC	Generation of pSA10-EspZ-2xHA-SBP
22R'	2xHA SBP tail R' GA	CCGGTGGTTTTTTCGTCCATACTTCCACCGCTTCCCCC	Generation of pSA10-EspZ-2xHA-SBP
23	Tags gblock	TATCGGTACTGGTATCGCAGCAATGGGAGGTTCTGGCGGGAGCTATCCATACGATGTGCCTGATTATGCGTACCCCTATGATGTGCCGGATTACGCGGGGGGAAGCGGTGGAAGTTGGAGTCATCCGAATTTCCAAAAGGGTGGCAGCGGTGGAAGCTGGAGCCACCCAAACTTTCAGAAAGGTGGCAGTGGAGGCAGCTTAGGCTTAGGAATCGCAGCCGGTG	Generation of pSA10-EspZ-2xHA-SBP and pSA10-EspZ-2xHA
