# Supplementary material for: Enteropathogenic E. coli infection co-elicits lysosomal exocytosis and lytic host cell death
Source: mBio. 2023 Dec 1;14(6):e01979-23. doi: 10.1128/mbio.01979-23 (PMC10746156; doi:10.1128/mbio.01979-23)
Supplement: Table S4 — Antibodies. [file mbio.01979-23-s0006.pdf]

Table S4: Primary and secondary antibodies

Antibody	Primary/Secondary dilution (IF/WB)	Description
Rabbit anti-Cathepsin D antibody	Primary 1:5000 (WB)	Rabbit monoclonal anti Cathepsin D antibody [EPR3057Y], ab75852; Abcam
Mouse anti-α-tubulin	Primary 1:2000 (WB)	αα-tubulin; Monoclonal anti-Tubulin-α antibody, Clone B512; T6074; Sigma-Aldrich
Mouse anti-Lamp1	Primary 1:2000 (IF)	Mouse monoclonal anti Lamp 1 antibody [H4A3], DSHB
Rabbit anti-FLAG antibody	Primary 1:1000 (WB)	Polyclonal anti-FLAG antibody; F7425; Sigma Aldrich
Mouse anti-β actin	Primary 1:5000 (WB)	Monoclonal antibody, ACTN05 (C4); #ab3280; Abcam
Mouse anti-HA	Primary 1:500 (WB)	αHA; Mouse monoclonal antibody (clone 12CA5) directed against the 9-amino acid sequence derived from the influenza hemagglutinin (HA) protein; #ab16918; Abcam
Rabbit anti-HA	Primary 1:5000 (WB)	Rabbit polyclonal anti-HA tag antibody - ChIP Grade; Abcam #ab9110

Rabbit anti-HA	Primary<br>1:800 (IF)	Rabbit monoclonal anti HA (C29F4), Cell Signaling Technology, (Danvers, MA, USA); Cat #3724
Donkey anti-Rabbit	Secondary<br>1:300 (IF)	Alexa Fluor 488- donkey anti- Rabbit IgG; Invitrogen (Molecular Probes); A21206.
Goat anti-mouse IgG, Alexa Fluor 488	Secondary<br>1:300 (IF)	Alexa Fluor 488- AffiniPure Goat Anti-Mouse IgG; 115-545-062; Jackson ImmunoResearch Laboratories
Peroxidase goat anti-mouse IgG	Secondary<br>1:10000 (WB)	Peroxidase- AffiniPure Goat anti-Mouse IgG; #115-035-166; Jackson ImmunoResearch Laboratories
Peroxidase goat anti-rabbit IgG	Secondary<br>1:10000 (WB)	Peroxidase- AffiniPure Goat Anti-Rabbit IgG; #111-035-003; Jackson ImmunoResearch Laboratories
