# Supplementary material for: Enteropathogenic E. coli infection co-elicits lysosomal exocytosis and lytic host cell death
Source: mBio. 2023 Dec 1;14(6):e01979-23. doi: 10.1128/mbio.01979-23 (PMC10746156; doi:10.1128/mbio.01979-23)
Supplement: Table S5 — Nikon TI filters. [file mbio.01979-23-s0007.pdf]

Table S5: Excitation and emission filters used for fluorophores imaged on the Nikon Ti microscope. Filters specified as center-wavelength/bandwidth.

Fluorophore	Chroma CAT	Excitation Filter	Emission Filter	Lowpass Filter
DAPI	49000 - ET - DAPI	AT350/50x V2	ET460/50m	T400lp
AlexaFluor488	49002 - ET - EGFP (FITC/Cy2)	ET470/40x	ET525/50m	T495lpxr
Propidium Iodide	49004 - ET - CY3/TRITC	ET545/25x	ET605/70m	T565lpxr
Phalloidin-CF647	49006 - ET - Cy5	ET620/60x	ET700/75m	T660lpxr
