# Supplementary material for: Enteropathogenic E. coli infection co-elicits lysosomal exocytosis and lytic host cell death
Source: mBio. 2023 Dec 1;14(6):e01979-23. doi: 10.1128/mbio.01979-23 (PMC10746156; doi:10.1128/mbio.01979-23)
Supplement: Table S6 — Nikon TI-E filters. [file mbio.01979-23-s0008.pdf]

Table S6: Excitation bands and emission filters used for fluorophores imaged on the Nikon Ti-E microscope. Filters specified as center-wavelength/bandwidth.

Fluorophore	Excitation Spectra X	Emission Filter Wavelength (Bandwidth)
DAPI	395/25 nm	435/26 nm
AlexaFluor488	440/20 nm	515/30 nm
Propidium Iodide	550/15 nm	595/40 nm
Phalloidin-CF647	640/30 nm	705/72 nm
